# Development of a novel mycobiome diagnostic for fungal infection

**DOI:** 10.1186/s12866-024-03197-5

**Published:** 2024-02-19

**Authors:** Danielle Weaver, Lilyann Novak-Frazer, Maisie Palmer, Malcolm Richardson, Mike Bromley, Paul Bowyer

**Affiliations:** 1 Core Technology Facility, University of Manchester, Manchester, M13 9WU UK; 2 Manchester University NHS Foundation Trust, Manchester, UK

**Keywords:** Mycobiome, Fungal infection, Diagnostics, Amplicon sequencing

## Abstract

**Background:**

Amplicon-based mycobiome analysis has the potential to identify all fungal species within a sample and hence could provide a valuable diagnostic assay for use in clinical mycology settings. In the last decade, the mycobiome has been increasingly characterised by targeting the internal transcribed spacer (ITS) regions. Although ITS targets give broad coverage and high sensitivity, they fail to provide accurate quantitation as the copy number of ITS regions in fungal genomes is highly variable even within species. To address these issues, this study aimed to develop a novel NGS fungal diagnostic assay using an alternative amplicon target.

**Methods:**

Novel universal primers were designed to amplify a highly diverse single copy and uniformly sized DNA target (*Tef1*) to enable mycobiome analysis on the Illumina iSeq100 which is a low cost, small footprint and simple to use next-generation sequencing platform. To enable automated analysis and rapid results, a streamlined bioinformatics workflow and sequence database were also developed. Sequencing of mock fungal communities was performed to compare the *Tef1* assay and established ITS1-based method. The assay was further evaluated using clinical respiratory samples and the feasibility of using internal spike-in quantitative controls was assessed.

**Results:**

The *Tef1* assay successfully identified and quantified *Aspergillus, Penicillium, Candida, Cryptococcus, Rhizopus, Fusarium* and *Lomentospora* species from mock communities. The *Tef1* assay was also capable of differentiating closely related species such as *A. fumigatus* and *A. fischeri.* In addition, it outperformed ITS1 at identifying *A. fumigatus* and other filamentous pathogens in mixed fungal communities (in the presence or absence of background human DNA). The assay could detect as few as 2 haploid genome equivalents of *A. fumigatus* from clinical respiratory samples. Lastly, spike-in controls were demonstrated to enable semi-quantitation of *A. fumigatus* load in clinical respiratory samples using sequencing data.

**Conclusions:**

This study has developed and tested a novel metabarcoding target and found the assay outperforms ITS1 at identifying clinically relevant filamentous fungi. The assay is a promising diagnostic candidate that could provide affordable NGS analysis to clinical mycology laboratories.

**Supplementary Information:**

The online version contains supplementary material available at 10.1186/s12866-024-03197-5.

## Introduction

Fungal infections affect over a billion people and kill an estimated 1.5 million annually [1]. The World Health Organisation recently defined *Aspergillus fumigatus, Candida albicans, Cryptococcus neoformans* and *Candida auris* as the fungal pathogens of highest public health importance [2]. Over 300 human fungal pathogenic species are known [3], although the actual number may be 5-fold higher [4] as the list of emerging fungal pathogens is continually expanding and includes pathogens such as *Candida auris* and species of *Fusarium*, *Mucor* and *Scedosporium* [5]. This range of possible fungal pathogens means that diagnosis using classical mycological techniques or single species PCR can be challenging.

Diagnosis of fungal infection requires consideration of several factors including culture, molecular assays, serology, radiology, and histopathology. Nevertheless, culture and microscopy remain the gold standard and diagnostic laboratories often rely on traditional mycology for identifying less common fungi such as Mucorales [6]. However, culture can be ineffective and time consuming, and microscopic identification requires significant technical expertise which may not be available [7]. Targeted molecular assays are now available which identify common fungal pathogens such as *Candida* and *Aspergillus*, and the latest EORTC/MSGERC consensus definitions for invasive fungal disease now includes the use of fungal PCR [8]. There also several broad-spectrum fungal biomarker tests such as beta-D glucan and galactomannan testing which are not species-specific [6]. More recently, point of care devices are coming to market which allow rapid identification of fungal pathogens such as *Cryptococcus* and *Aspergillus* [9]. However, antigen-based methods may not identify the pathogen to species level and may be prone to false positives [10]. In addition, although species-specific assays exist, many available PCR assays provide only genus-level identification and/or may have variable sensitivity for emerging or less commonly isolated species, as demonstrated for *Aspergillus* [11]. Identification to the species level is desirable for a diagnostic as it can often be informative for treatment decisions. For example, amphotericin B may not be suitable when treating an *Aspergillus flavus* infection, as there are several reports of resistance and treatment failure with this pathogen [12].

DNA sequencing of hypervariable regions can add significant value to diagnostics, allowing isolates to be identified by comparison of sequences to an established database. Targets used for fungal identification include ribosomal RNA, internal transcribed spacer (ITS) regions, beta-tubulin, calmodulin and translation elongation factor alpha (TEF) [13]. Sanger DNA barcoding for pan fungal identification and speciation from complex samples first requires culture of an organism as this method alone cannot resolve the numerous amplicon variants that pan fungal PCR may produce if different fungi are present. This limits the sensitivity and usefulness of this method to culturable organisms that grow within the defined culture conditions. An improvement on this method is the use of next-generation sequencing (NGS) which can sequence the marker gene of an entire community (metabarcoding). McTaggart et al. [14] demonstrated the applicability of mycobiome sequencing for complementing diagnosis of infections caused by the endemic fungal pathogen, *Blastomyces dermatitidis*. This study also noted the identification of other fungal pathogens in culture-confirmed samples, highlighting the potential for broad coverage of this method.

Mycobiome studies to date use ribosomal internal transcribed spacer (ITS) regions ITS1 or ITS2. However, the copy number of the rDNA region varies up to 13-fold within a population of *A. fumigatus* isolates [15] and, broadly, varies up to 100-fold in the fungal kingdom [16]. Therefore, quantitative PCR targeting ITS regions is unlikely to accurately quantify species abundance, even in the presence of DNA standards. Varying copy number may introduce bias when determining the relative abundance of species in a community and a single copy gene may offer an improved estimation. Single copy genes previously used as molecular barcodes for Fungi include beta-tubulin, calmodulin, and translation elongation factor 1-alpha (*TEF1a*). Targeting these genes can often provide a higher species resolution compared to ITS regions, however, it is difficult to generate universal fungal primers with high performance [13].

An ideal DNA barcode would be able to delineate different species both within a genus and between genera [17]. High intra-species variation within the ITS regions can reduce this barcode gap and in turn the resolution power, as is the case with *Penicillium* and *Fusarium* species [13]. This extensive variation can also complicate analysis, requiring the curation of a larger molecular database.

Existing mycobiome techniques and databases have been established to analyse community structure. The advantage of NGS amplicon analysis is the ability to identify many species in a single reaction from a single sample and detailed knowledge of the community composition is not always needed. We set out to develop a system that retained the advantages of microbiome analysis but that was focused on accurate detection of important species in a simplified NGS workflow. Given the known issues with ribosomal RNA targets, we sought to develop a metabarcoding assay which targeted a single copy gene, to potentially enable reference standards to be used to establish a quantitative sequencing diagnostic. Here we describe the design and validation of a fungal metabarcoding-based diagnostic assay based on an amplicon within the *TEF1a* gene which exhibits less intra-species variation within fungal species, including *Penicillium* and *Fusarium*, and is therefore better able to discriminate between species [13, 18]. In addition, we demonstrate a proof-of-principle using spike-in controls to achieve semi-quantitative pathogen identification from clinical samples.

## Methods

### Clinical samples

All clinical samples used in this study were anonymised residual DNA extracts sourced from Manchester University NHS Foundation Trust, Wythenshawe. Samples were a variety of residual respiratory DNA extracts previously used for *Aspergillus* PCR screening performed during routine clinical duties. The Health Research Authority system [19] was used to assess this study and confirmed that ethical review was not a requirement.

### *In silico* PCR analyses

*in silico* PCR was performed using PrimerTree v1.0.5 [20]. Primer search parameters were 50 permutations, 1000 alignments and 3 mismatches. Searches were performed with and without ‘Fungi’ organism filter. For TEF searches, the two primer sets were collapsed to form a single representative primer pair. Prior to plotting, product lengths were filtered to be below 800 nt. For creating species level phylogenetic trees, sequences were retrieved from NCBI nt/nr database (https://www.ncbi.nlm.nih.gov/nuccore), trimmed to the specific amplicon, aligned using MUSCLE and neighbour-joining trees generated using the nucleotide difference method in MEGA X [21].

### Construction of fungal sequence database

Fungal sequences were obtained using NCBI Blast nonredundant nucleotide database. Duplicated sequences, sequences containing non-nucleotide letters, that were over 2000 nucleotides (nt) or less than 200 nt, were removed using a modified version of Sequence Cleaner (Biopython) [22]. Sequences that included both primer sequences only were maintained. Manual curation of the database was applied throughout the process, although a specific set of 50 common human fungal pathogens were purposefully curated using a combination of self-mapping tests using bowtie2 [23] and further searches against NCBI WGS database and ISHAM TEF barcoding database (https://its.mycologylab.org) [24]. A comprehensive curation of *Aspergillus*, *Penicillium* and *Fusarium* was performed to define sequences within closely related species complexes. During this, additional sequences were mined from genome data sourced from JGI Mycocosm (https://mycocosm.jgi.doe.gov) and Ensembl Fungi (http://fungi.ensembl.org). Where available, sequences were added from in-house sequencing of laboratory isolates (termed with “_MFIG” suffix in sequence headers). For *Aspergillus* speciation comparison trees, *Aspergillus* sequences were extracted and primer regions were removed prior to alignment using clustal to generate maximum likelihood trees (500 bootstraps, Tamura Nei model) within MEGAX. To create the corresponding ITS1 tree, *Aspergillus* ITS1 sequences were extracted from ISHAM ITS and UNITE (https://unite.ut.ee/repository.php) databases.

### Fungal culture and DNA extractions

Filamentous fungi were grown on Sabouraud (SAB) agar for 48 h at 30℃ (37℃ for *A. fumigatus*). Spores were harvested in PBS 0.1% Tween and washed in milliQ water. Cells were lysed mechanically using cetyl trimethyl ammonium bromide (CTAB) buffer (Generon Ltd), glass beads (425–600 μm, Sigma) and a Precellys 24 homogeniser (Bertin Technologies). Supernatants were treated with RNase A (Qiagen) and DNA extracted using chloroform and ethanol precipitation. Yeasts were grown in potato dextrose broth at 30℃ for up to 24 h, harvested in PBS and washed in mQ water. Cell pellets were lysed mechanically using CTAB buffer (plus 0.1 mg/ml proteinase K and 0.2% beta-mercaptoethanol) and vortexing with glass beads (*C. neoformans* was subject to an additional homogenisation cycle). For *A. clavatus, F. oxysporum, N. crassa, R. arrhizus* and *S. prolificans*, liquid SAB cultures were incubated at 30℃ until formation of a mycelial mat. Mycelia were pressed and air dried at room temperature. Dried mycelia were lysed using cycles of snap freeze (using liquid nitrogen) and vortexing with glass beads (4 mm, Sigma) and an SDS lysis buffer (200 mM Tris-HCl, 1% SDS, 500 mM NaCl, 100 mM EDTA). DNA was extracted using phenol chloroform and isopropanol precipitation [25]. Samples were treated with RNase A and subject to a final ethanol purification. Human DNA was extracted from an A549 cell line using DNeasy blood and tissue kit (Qiagen). DNA quality was checked using 260/280 nm absorbance ratios on a Nanodrop spectrophotometer (ThermoFisher Scientific) and quantified using the Qubit dsDNA HS assay kit (ThermoFisher Scientific).

### PCR

For initial analyses, amplicon PCR reactions were performed with Phusion Green Hot Start II High-Fidelity PCR Master Mix (ThermoFisher Scientific) and index PCR reactions with KAPA HiFi HS RM (Roche). Following optimisation with clinical samples, KAPA HiFi HS RM was used for both PCR reactions. Primers are described in Table S1. All PCR reactions were checked by agarose gel electrophoresis. Amplicon PCR reaction volumes were 25 µl containing 2 µl DNA. ITS1 amplicon PCRs included 0.3 µM FunITS1-Ill and 1.2 µM FunITS2-Ill. Cycling conditions were: 98℃ for 30 s, 30 cycles of 98℃ for 10 s, 55℃ for 30 s and 72℃ for 15 s and a final extension for 10 min at 72℃. TEF amplicon PCRs included 0.4 µM of the NGS-compatible forward primer mix and 1.5 µM of the NGS-compatible reverse primer mix. TEF NGS-compatible primers have the prefix “TEF_Nx_” (see Table S2). In addition, standard TEF primer mixes (lacking the Nextera adaptors) were added to the reaction at 40 nM and 150 nM for forward and reverse primer mixes, respectively. This small addition of standard primers into the PCR was tested against gDNA of *A. fumigatus* and *C. albicans* and gave increased amplicon yield (data not shown). TEF primer mixes were created using the following ratios to reflect the possible primer combinations within each redundant primer: forward mix contained 1:0.5 of F2_1b:F2_2b, reverse mix contained 1:3.7 of R2_1b:R2_2b. Cycling conditions were: 98℃ for 30 s, 20 cycles of 98℃ for 10 s, 64℃ for 30 s and 72℃ for 12 s, 20 cycles of 98℃ for 10 s, 70℃ for 30 s and 72℃ for 12 s and a final extension for 10 min at 72℃. Following clinical optimisation of the PCR parameters, an annealing temperature of 67 ℃ and 50 cycles were chosen. As TEF PCR reactions were uniquely barcoded, products were pooled immediately following amplicon PCR.

### Sequencing library preparation

Samples were processed as per the Illumina fungal metagenomic demonstrated protocol (Starting at ‘Clean up’ section) using Nextera XT index kit v2 set A. However, this protocol was modified so that ITS1 products were pooled prior to ‘Clean up 2’ and, for the final library pooling step, products were pooled according to the number of samples they represented. The final library pool was diluted to the required dilutions and loaded onto the iSeq100 and MiSeq sequencing instruments (Illumina). Sequencing reagent kits used were iSeq100 i1 and MiSeq Micro kit v2 (300 cycles).

### Sequencing data analysis

For TEF data, paired end reads were first merged using bbmerge from bbmap v38.22 [26]. Samples were demultiplexed using FastX-Toolkit v0.0.14 (http://hannonlab.cshl.edu/fastx_toolkit.). Reads were quality trimmed at q30, filtered by a minimum length of 150 nucleotides (nt), and primer sequences were removed using Cutadapt v1.18 [27]. ITS1 sequencing data were processed without a read merge step, as some amplicons are too larger to be merged successfully. Paired end reads were subject to quality trimming at q30 and filtered by a minimum length of 75 nt using bbduk from bbmap. Primer sequences were removed using Cutadapt.

End-to-end alignments were performed using Bowtie2 v2.3.5.1 [23]. TEF merged reads were mapped to an in-house curated database of TEF sequences. ITS1-paired end reads were mapped to the ISHAM ITS database. (Species counts were extracted from alignments using SAMtools v1.9 [28]. Species counts were processed using custom-made shell and R scripts. Briefly, species present below a 0.2% cut-off were removed from a sample and relative proportions calculated for the remaining species. Samples were plotted alongside expected mock communities using ggplot2 v 3.3.2 [29] and stringr v 1.4.0 [30] in Rstudio v1.2.1578 (R v 3.6.3) [31, 32]. To produce abundance plots with dendrograms, species counts were processed using phyloseq v1.30.0 [33]. Data were normalised and subject to a minimum 0.2% abundance taxa filter. Bray-Curtis distances were calculated and subject to hierarchical clustering to create dendrograms with ggdendro [34]. As described previously, plots were generated with ggplot2.

For combined analysis of all mock community samples, only the corresponding expected species were retained per sample. Relative species proportions were recalculated, and Chi squared tests were performed in R. Species identification rates were calculated and plotted using pheatmap v1.0.12 [35] in R. Species percent abundance statistical analyses were performed using dplyr v1.0.0 [36], tidyr v1.1.0 [37] and ggpubr v0.3.0 [38].

### Quantitative PCR

Quantitative PCR (qPCR) was performed on an Applied Biosystems 7500 FAST system using *Power* SYBR® Green PCR Master Mix (Applied Biosystems) in standard cycling mode as per manufacturer’s instructions. Each forward primer was used at 0.4 µM with 3.7X and 4X of TEF reverse primer mix and Fun_ITS2, respectively. Standard curves were comprised of 50,000 haploid genome equivalents of DNA and a further four 5-fold serial dilutions (For *C. albicans, A. fumigatus* and *L. prolificans*). For the 21 species comparison experiment, 50,000 genome copies were used. Each qPCR experiment was performed in triplicate and are presented together with standard deviation. Statistics (2-way ANOVA, ANOVA and Tukey’s multiple comparisons tests) were performed on Ct values in GraphPad Prism v9.

Primers pC[1,4 or 5]_F and pC[1,4 or 5]_R were used at 0.2 µM for qPCR to validate the concentration of internal plasmid control (IPC)[1,4 or 5] within spike-in DNA mixes. Standard curves consisted of a 10-fold serial dilution series of each IPC ranging between 2 and 20,000 copies per reaction.

For validation of *A. fumigatus* levels in mock and clinical respiratory samples, a TaqMan probe assay targeting the ITS1 region was used [39]. Assays were performed with TaqMan Fast Advanced Master Mix (ThermoFisher Scientific). Primers and probe are listed in Table S1 and were added at 750 nM and 300 nM, respectively. Cycling parameters were 50℃ for 2 min, 95℃ for 2 min, 50 cycles of 95℃ for 3 s and 60℃ for 30 s. Standard curves consisted of a nine sample 10-fold dilution series starting with 100 ng *A. fumigatus* haploid genome equivalents (GE) per reaction.

### Spike-in controls

Amplicons to be used as spike-in control DNA were designed by scrambling known fungal TEF sequences (primer sequence regions were not scrambled) and synthesising the DNA in a pEX-A128 vector (Eurofins Genomics, GmbH). Plasmid DNA was quantified using Qubit dsDNA HS assay kit to allow copy number calculations. To create spike-in standards, plasmid DNA was then diluted and mixed as appropriate. The copy number of each plasmid control was validated using qPCR.

## Results

### Design and in silico evaluation of a novel fungal metabarcoding assay

Extant DNA metabarcoding techniques for speciating and quantifying fungal populations are often considered inadequate as significant biases are generated during PCR amplification and sequencing. To address this and to explore the possibility of developing a clinical mycobiome diagnostic, we designed a series of novel universal primers that target the Translation Elongation Factor 1-alpha (*TEF1a*). To design this target, *TEF1a* sequences from 30 clinically relevant fungal pathogens (including *Aspergillus, Candida, Cryptococcus, Malassezia, Pneumocystis, Rhizopus*, *Scedosporium* and *Trichophyton*) were aligned to identify a conserved region suitable for creating a small amplicon (˜ 250 bp) that had uniform size between species and with good speciation capability. Although the site displaying greatest variability was flanked by semi-conserved sequences, the level of conservation was not sufficient to allow us to design a single degenerate primer pair. To overcome this we developed two degenerate primer sets at this site, in essence creating a multiplex assay. The two primer sets broadly target either filamentous fungi or yeast, although not exclusively (see Fig. S1A). Throughout this study, the performance of the TEF multiplex primer set and metabarcoding assay was compared to a modified version of the original fungal ITS1 primers, ITS1 and ITS2 [40], which have been optimised in our laboratory to allow amplification of the common respiratory fungal pathogen, *Pneumocystis jirovecii* (see Table S1). This was achieved by introducing redundancy into the reverse primer, ITS2, to generate an optimised version termed FUN_ITS2_1.

To predict the taxa coverage of the ITS1 and TEF metabarcoding primer sets, *in silico* PCR was carried out against the NCBI nucleotide sequence database using PrimerTree [20] (Fig. S2A). The ITS1 products generated in this evaluation comprised amplicons from kingdom Fungi (33%), Metazoa (48%) and Viridiplantae (19%). Most non-fungal hits were from the phyla Cnidaria (50%) and Streptophyta (28%). The Streptophyta hits included common food-related species, which could be present in human gastrointestinal or respiratory tract samples, such as mustard, cabbage and barley. Plant amplification is a known feature of the original ITS1 primers [41]. Alternatives designed to reduce this plant amplification have emerged since, however, they often generate a larger amplicon that is less suitable for short read sequencing [41]. The TEF primers amplified products from Fungi (48.5%), Metazoa (51.4%) and Viridiplantae (0.1%). Of the non-fungal TEF products, 94% were from the phylum Arthropoda. Regarding fungal specific coverage, each target had hits covering the fungal phyla that contain all known human pathogens; Ascomycota, Basidiomycota, Mucoromycota and Zoopagomycota. (Fig. S2B) [42]. The lack of hits from the remaining five fungal phyla (Opisthosporidia, Chytridiomycota, Neocallimastigomycota, Blastocladiomycota and Glomeromycota) [43] is likely due to bias in the availability of sequence data, rather than an inability of both primer sets to amplify these taxa, as the previously described phyla are significantly better studied.

**Fig. 1:**
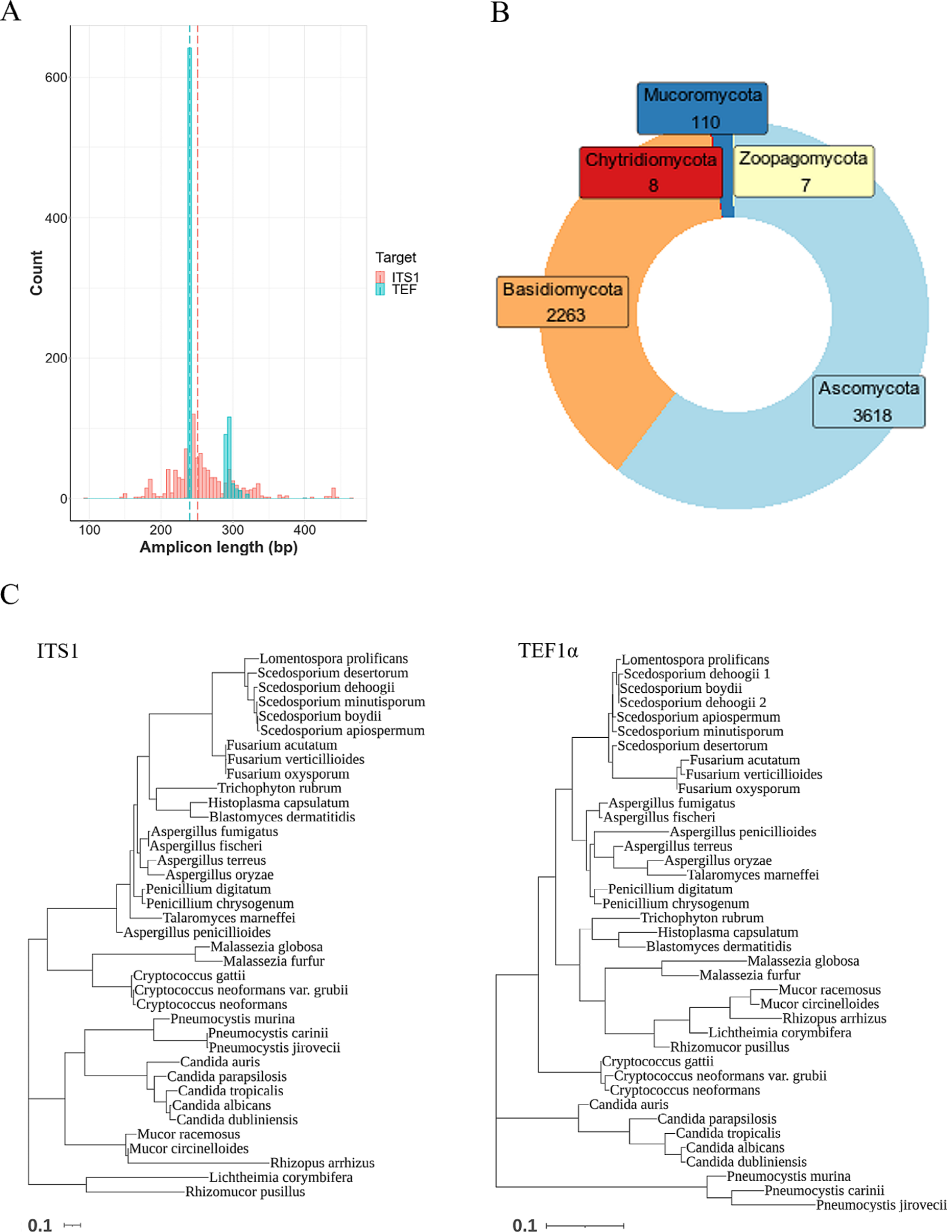
*In silico* analyses of ITS1 and TEF1α targets. (**A**) PrimerTree was used to search primers against fungal sequences within the NCBI nr nucleotide database. The frequency of product lengths are displayed for each target (vertical dotted line indicates median). (**B**) A curated database of TEF sequences was established. The 8656 sequences cover the five phyla containing known human pathogenic fungi and the number of species covered within these phyla are shown. (**C**) ITS1 (left) and TEF1α sequences were obtained for over thirty clinically relevant fungal species and alignments performed using Clustal to demonstrate fungal speciation. Phylogenetic trees were generated using maximum-likelihood method and Tamura-Nei model

Amplicon size is an important consideration when developing a metabarcoding strategy. Most NGS platforms are most cost efficient when sequencing smaller amplicons. Furthermore, if the amplicons targeted by a universal primer set are highly variable in size from species to species, biases can be introduced as smaller amplicons are preferentially amplified in PCR reactions. The size of many fungal ITS amplicons exceed the capability of many NGS sequencing platforms. Additionally, if forward and reverse reads from paired end sequencing are merged to produce overlapping reads non-overlapped pairs can be discarded in some analysis pipelines. To estimate the diversity of amplicon size for the two targets, PrimerTree searches were performed against fungal sequences from the NCBI nucleotide database. TEF amplicons were bimodal, the majority (71%) were 240 base pairs (bp) whereas a second smaller group, mostly (94%) belonging to Agaricomycetes, had amplicons from 286 to 321 bp (Fig. 1A). Not all Agaricomycetes have the larger amplicon though, and the only human pathogen within this class, *Schizophyllum commune*, produces a 240 bp amplicon. One species of soil-inhabiting yeast (*Tausonia* sp.) produced a 402 bp amplicon. Therefore, TEF amplicons of human fungal pathogens are both uniform in size and well suited to 2 × 150 sequencing, which can be performed on the affordable Illumina iSeq100. The median amplicon length for ITS1 was 251 bp, however, the amplicon length was much more variable, with amplicons ranging from 97 to 465 bp. This ITS1 amplicon variation is a problem for human fungal pathogens too, as the ITS1 sequences of our clinically relevant species set ranged between 201 and 346 bp. The significant amplicon length variation may affect ITS1 performance when using shorter sequencing strategies. Although other ribosomal RNA targets used for fungal profiling can display less size variation than ITS1, the mean length of described amplicons are often larger (e.g. over 300 bp for ITS2) [44] and are therefore less desirable for use with particularly short read technologies such as the iSeq100 instrument.

Sequences for over 30 clinically relevant fungal species (and some close relatives) were aligned to demonstrate the speciation capabilities of each amplicon. Representative sequences of each target were gathered using NCBI sequence databases (RefSeq sequences were chosen preferably, where possible). It was first confirmed that the TEF primer set was predicted to amplify all these sequences (> 75% of targets had no primer mismatches, remaining species had no more than 2 mismatches with any primer). Phylogenetic trees of these datasets demonstrate that, although less variable than ITS1 overall, the TEF amplicons can differentiate between most species assessed (Fig. 1C). For example, the amplicon sequence can distinguish *A. fumigatus* from the closely related species, *A. fischeri* (2 bp variants). The sequence of the human pathogenic *Pneumocystis* species (*jirovecii*) is separated from that of the murine infecting species *Pn. murina* (32 bp variants) [45]. In addition, *Cryptococcus gattii*, *C. neoformans* var *neoformans*, and *C. neoformans* var. *grubii* (the latter two species recently renamed *C. deneoformans* and *C. neoformans*, respectively) can be differentiated. One exception is within the *Scedosporium apiospermum* species complex, where there are unique sequences for each member except *S. boydii*. One of the two *Scedosporium dehoogi* sequences available is identical to the only available *S. boydii* sequence; whether this interspecies sequence crossover is true or a database inaccuracy is unclear, and more data are needed to resolve this. ITS1 can differentiate all the species assessed, excluding *Fusarium oxysporum* and *F. acutatum*. Therefore, TEF is predicted to amplify and provides similarly good identification of a variety of clinically relevant fungi.

To facilitate identification using the metabarcoding assay, we constructed and curated a database of TEF sequences using publicly available sequences from NCBI databases and/or extracted from available genomes. The current database contains ˜ 8600 TEF sequences from fungi belonging to 38 classes, covering over 1600 genera and over 6000 species (Fig. 1B).

To further illustrate the ability of each target to differentiate species, sequences of *Aspergillus* species obtained from ISHAM barcoding and UNITE databases for ITS1 and our in-house database for TEF were compared. Species were discarded if there were no representative sequences for both targets. Primer regions were removed from the sequences prior to alignment and generation of maximum likelihood trees. The resulting trees (Fig. S3) highlight some difficulties with *Aspergillus* speciation (coloured boxes highlight examples within the *A. fumigatus, A. flavus* and *A. niger* species complexes). For example, both targets are unable to differentiate some members of the *A. fumigatus* species complex (SC) (highlighted in red). Although *A. fumigatus* is distinct from other species within the SC, both targets are unable to differentiate some clinically relevant non-*fumigatus* species such as *A. lentulus*, *A. viridinutans*, and *A. novofumigatus* [46]. ITS1 cannot distinguish *A. fischeri* from this species set, whereas TEF can delineate this species from all others. In addition, ITS1 fails to resolve *A. niger* species complex (SC), with seven species appearing indistinguishable from *A. niger* (highlighted in blue). ITS speciation issues within this SC are noted in the literature [47]. However, there is also an apparent mis-annotated *A. niger* sequence clustering away from its true SC, instead situated close to *A. carbonarius* and *A. sclerotioniger* sequences. This outlier demonstrates the difficulties sometimes faced when using publicly available ITS1 sequence databases. In contrast, the new curated TEF database shows a clearer picture, with *A. niger* sequences clustering together with two other closely related members of the species complex (*A. niger* and *A. awamori*), with other members of the SC separated from these (Fig. S3, highlighted in blue). Lastly, both targets are unable to differentiate the clinically relevant *A. flavus* from its less pathogenic relative, *A. oryzae*. However, the overall species resolution within the *A. flavus* SC in ITS1 is also poor, with overlapping sequences for *A. flavus, A. parasiticus* and *A. minisclerotigenes* species and species outside of this SC also clustering together. This section also demonstrates the high intraspecies variation often found within ITS1 [18], as there are 25 variations of *A. flavus* sequences alone (highlighted in grey). In contrast, there are only two *A. flavus* TEF sequences.

### Amplification with the TEF fungal primers reduces the PCR bias observed with ITS1 primers

As the objective of this study was to develop an NGS compatible fungal assay, both the TEF primer sets were modified to incorporate Illumina Nextera adaptor sequences and dinucleotide spacers to avoid introducing amplification bias (Table S1). Furthermore, we included barcodes to allow multiplexing of up to 96 samples (see Table S2, Fig. S1B). Using these primers, we attempted to amplify PCR products from 19 fungal species, encompassing pathogenic species within *Aspergillus, Penicillium, Fusarium, Candida*, and *Cryptococcus.* For all species tested, a single amplicon of c. 320 bp was clearly amplified (Fig. S1C). Our assessment of the PCR assay to amplify from samples containing low copy numbers of target, revealed we could detect as few as 2 haploid genome equivalents (GE) of *A. fumigatus* and *C. albicans* (Fig.S1D). As human DNA is likely to be abundant in clinical samples, we tested the ability of the TEF primers to amplify human TEF from c. 15,000 haploid genome copies of human gDNA. *In silico* analysis revealed between 2 and 7 mismatches with human eEF1A gene sequences in the primer regions, and the primers were able to amplify a low abundance (˜ 5X lower than *A. fumigatus* amplicon) product at c. 240 bp but also an unknown, low abundance (˜ 6X lower than *A. fumigatus* amplicon) product of c. 600 bp (Fig. S1E).

**Fig. 2:**
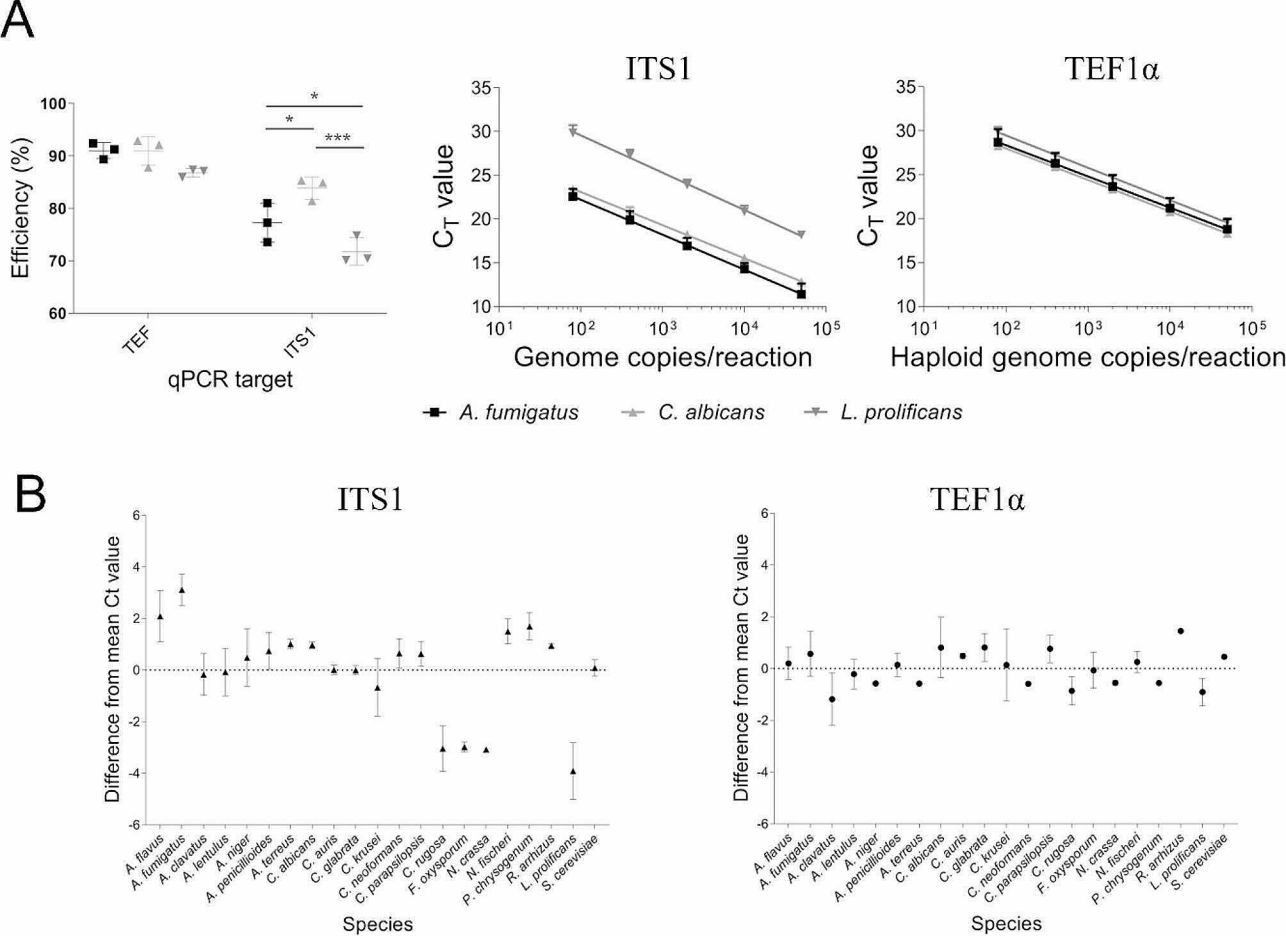
Fungal amplification using TEF primers is more efficient and displays reduced species bias compared to ITS1. A. Quantitative PCR efficiency (left) was significantly higher with TEF compared to ITS1 for all three species (*P* < 0.0001, 2-way ANOVA). Species efficiencies were significantly different from one another for ITS1 (*; *P* < 0.05, ***; *P* < 0.001 by 2-way ANOVA Tukey’s multiple comparisons). Relative quantitation of (**A**) *fumigatus, C. albicans* and *L. prolificans* using the ITS1 (middle) and TEF (right) PCR assays shows under-representation of *L. prolificans* with ITS1. (**B**) Quantitative PCR of 50,000 haploid genome equivalents of twenty one fungal species shows species quantitation is more variable with ITS1 than TEF. Data is visualised as difference from mean Ct value. Ct values were significantly different between species for TEF and ITS1 (*P* < 0.001 and *P* < 0.0001, respectively, by ANOVA. Of the 210 Tukey’s multiple comparisons tests performed, 6 and 81 were significant for TEF and ITS1, respectively (*P* < 0.05, not indicated)

To evaluate the efficiency of target amplification using the TEF and ITS1 primer sets, genomic DNA from three fungal species were used to create quantitative PCR (qPCR) standard curves for each assay. The TEF assay had significantly higher efficiency compared to ITS1 (Figs. 2A and 89.5% vs. 77.4%, *P* < 0.0001, Two-way ANOVA). When individual species (*A. fumigatus, C. albicans* and *L. prolificans*) were compared, a significant difference was observed between efficiencies for ITS1 (*P* < 0.05, Tukey’s multiple comparisons) but not TEF. Amplification efficiency of ITS1 for *L. prolificans* was significantly lower than all the other species (*P* < 0.001) however this was not the case for TEF, where amplification efficiency was not significantly different between species. To ensure the modified version of the reverse ITS1 primer (FUN_ITS2_1) was not the cause of the variation in amplification efficiency, we also performed this experiment with the original ITS1 primer pair (ITS1 and ITS2) and had similar results (Fig. S4). This relative difference in *L. prolificans* quantitation with ITS1 may be because this species has two mismatches to the ITS1 forward primer (Fig. 2A).

To investigate relative species quantitation using the TEF amplicon further, qPCR was performed with c. 50,000 haploid genome equivalents (GE) of twenty-one fungal species using each target. Ct values were significantly different between species for both targets (*P* < 0.001 for TEF, *P* < 0.0001 for ITS1). The relative quantitation using the TEF primer set was more consistent when compared to the ITS1 primer set (81 and 6 Tukey’s multiple comparisons tests significantly different for ITS1 and TEF, respectively (*P* < 0.05); Fig. 2B). In particular, we noted that relative quantitation of *A. fumigatus*, *C. rugosa, F. oxysporum, L. prolificans* and *N. crassa* with ITS1 showed a ˜ 3–4 Ct value difference from the other fungal species.

### Sequencing of mock communities shows the TEF metabarcoding assay outperforms the ITS1 assay

We performed pilot sequencing of mock fungal communities using Illumina iSeq100, to compare the performance of TEF and ITS1 amplicons in an NGS assay. Mock communities contained genomic DNA from between five and eight fungal species, each containing a predominance of DNA from one species with the remaining members of the mock community spiked at 2% haploid genome equivalents. TEF sequencing data were aligned to our curated TEF database using Bowtie2. The ITS1 data were aligned to the ISHAM ITS database containing ˜ 4,000 ITS sequences from 621 fungal pathogens.

**Fig. 3:**
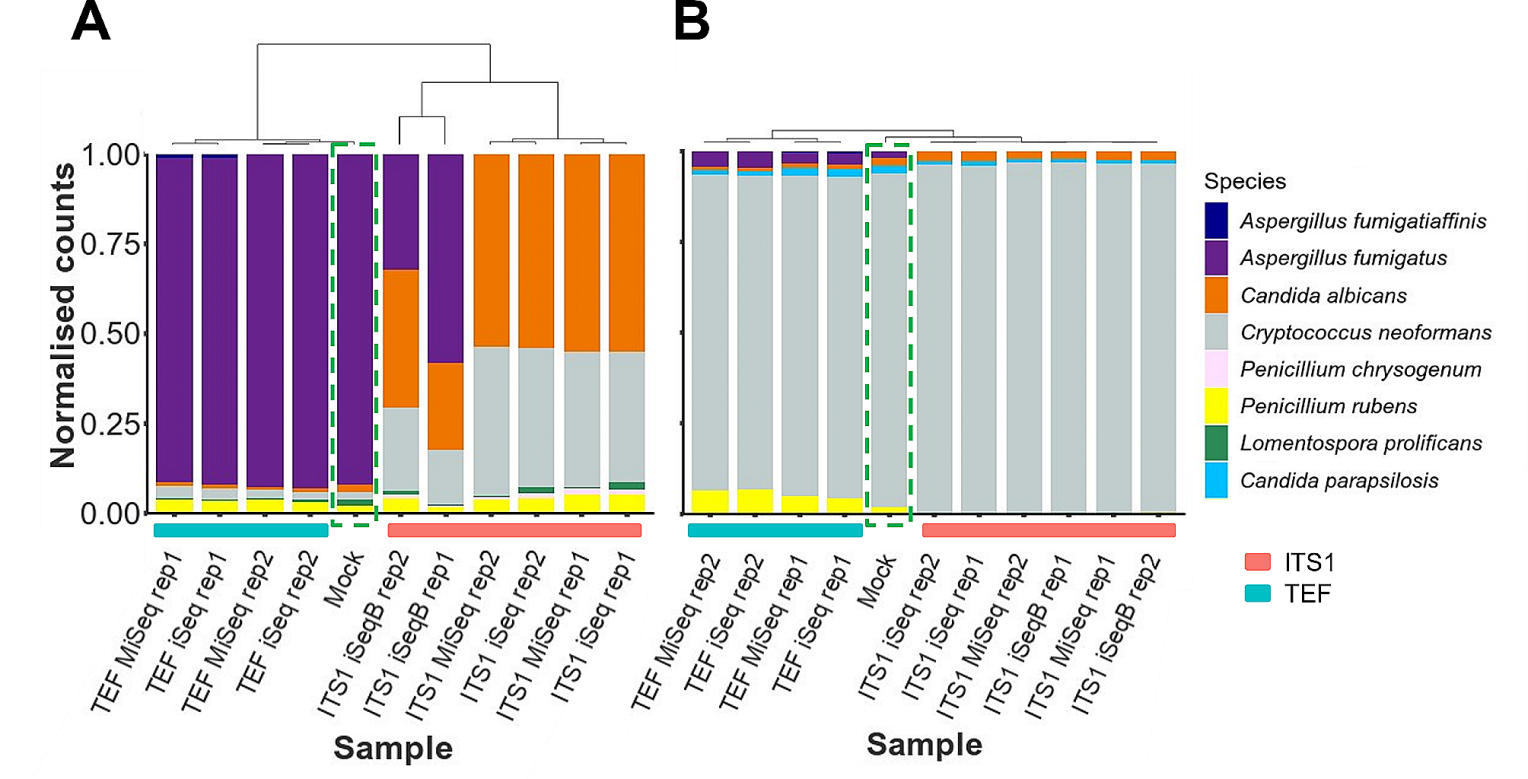
TEF assay outperforms ITS1 when sequencing fungal mock communities. Two representative fungal mock community analyses are shown. Each community contained 5 species, was targeted by TEF and ITS1 in duplicate and sequenced on an Illumina iSeq and MiSeq. For ITS1 samples, iSeq sequencing was performed twice. Bar plots indicate normalised species count data and expected mock community (dashed green box). Dendrograms indicate hierarchical clustering of Bray-Curtis distances and samples are ordered accordingly. A. ITS1 either significantly under-estimates or fails to identify (**A**) *fumigatus* when it is dominant (92% genome equivalents) in a community. TEF identifies all species present and at close to the expected relative abundances. (**B**) ITS1 correctly identifies (**C**) *neoformans* when it is dominant but fails to identify filamentous species present at a low level (*A. fumigatus* and *P. rubens).* TEF identified all species present and at close to the expected relative abundances

In total 14 mock communities were compared using the ITS1 and TEF assays. Consistent with the results of the PCR bias we observed against *A. fumigatus* using the ITS1 primers in a single species DNA sample (Fig. 2B), it was clear that *A. fumigatus* was not efficiently detected in the sequencing data. For example, mock communities created with a high proportion (92% GE) of *A. fumigatus* DNA that contained 2% *C. albicans* DNA were consistently identified as being dominated by *C. albicans* (See representative data in Fig. 3A and corresponding raw count data in Fig. S5). In contrast, TEF was able to correctly assign *A. fumigatus* as the dominant species in these samples (Fig. 3A). A similar outcome was observed when analysing mock communities with *P. rubens* as the dominant species. This poor relative detection of *A. fumigatus* using ITS1 primers was evident in three independent sequencing experiments, all assessing mixes with the same relative proportions of species and performed on the iSeq100. Moreover, we found the same issue when these samples were sequenced on a MiSeq using a 2 × 150 Micro V2 kit (Fig. 3A). Bray-Curtis distances were used to cluster expected community and sample data. All ITS1 samples clustered separately from TEF and the expected community, with ITS1 and TEF mock distances ranging between 0.92 and 0.94 and 0.02–0.04, respectively. Within the ITS1 and TEF expected clusters, sample replicates sequenced by either instrument clustered together, indicating that there was no clear evidence of sequence platform related bias.

When analysing a mock community dominated by *C. neoformans*, (with *C. albicans, C. parapsilosis, A. fumigatus* and *P. rubens* each at 2%), both targets correctly identified *C. neoformans* as the most abundant species (Fig. 3B). Bray-Curtis distances were very small (< 0.08) for all sample outcomes, regardless of target. The TEF assay correctly identified all species at levels consistent with the input ratios. However, although the ITS1 assays correctly assigned both *Candida* species, *A. fumigatus* and *P. rubens* were not identified. As *C. albicans* is not over-represented in this *Cryptococcus*-high mix, this suggests the striking outcome in the previous *A. fumigatus-* and *P. rubens*-high mixes is due to a bias between the representation of yeast and filamentous fungi in the species set analysed.

The inability of the ITS1 assay to detect *A. fumigatus* is most evident when present at low copy number in a mixed sample, in contrast to experiments assessing the limit of detection (LOD) for the assays, where both targets were able to detect as few as 2 copies of *A. fumigatus, P. rubens* and *C. neoformans* when sequenced in isolation (Fig. S6). However, ITS1 read counts for *C. neoformans* were around 6-fold higher than those for *A. fumigatus* when present in samples with equal genome equivalents; in contrast, only a ˜ 0.5-fold variation was seen with TEF. This aligns with our previously unpublished observations made using an IonTorrent sequencer, that *A. fumigatus* ITS1 sequences were specifically under-represented when performing amplicon sequencing on gDNA of various species in isolation.

Notably, in one of each duplicate set in Fig. 3, TEF identified a small amount of an unexpected species, *A. fumigatiaffinis* (1.2% or less). This species is closely related to *A. fumigatus* and its presence could be due to misidentification. Rather, this event is likely due to a contamination event, as single species samples containing between 2 and 100 copies of *A. fumigatus* do not produce this unexpected species (Fig. S6D). Such instances of contamination, although not species specific, were sometimes identified in other TEF mock community samples within the experiment. Contaminating species were generally found at a low level with median percent abundance of 0.8%. Contaminating events were likely due to either sample or primer crossover during sample preparation. Either cause is a particular risk when working with mock community samples due to the high genome copy numbers present. There was relatively little contamination in ITS1 data, suggesting it is due to TEF barcoded primer crossover. In line with this, contamination when preparing high copy number single species samples was eliminated by use of newly purchased barcoded primers and increased care with respect to contamination prevention during solution handling (Fig. S7). For simplicity, apparent contamination events were removed from the data before performing further analyses.

To compare the ability of ITS1 and TEF to quantify relative proportions of species within mixes, Chi^2^ tests were applied to sequenced mock community samples and the corresponding expected species proportions. For samples analysed using ITS1, 8/19 and 10/22 of samples were significantly different from the expected input community for iSeq and MiSeq instruments, respectively (*P* < 0.05, see Table S3). All significantly different samples contained either *A. fumigatus* or *P. rubens* as the dominant species. Levels and species composition of mock samples analysed using TEF primers showed no significant difference from the expected input mock communities (Table S3).

**Fig. 4:**
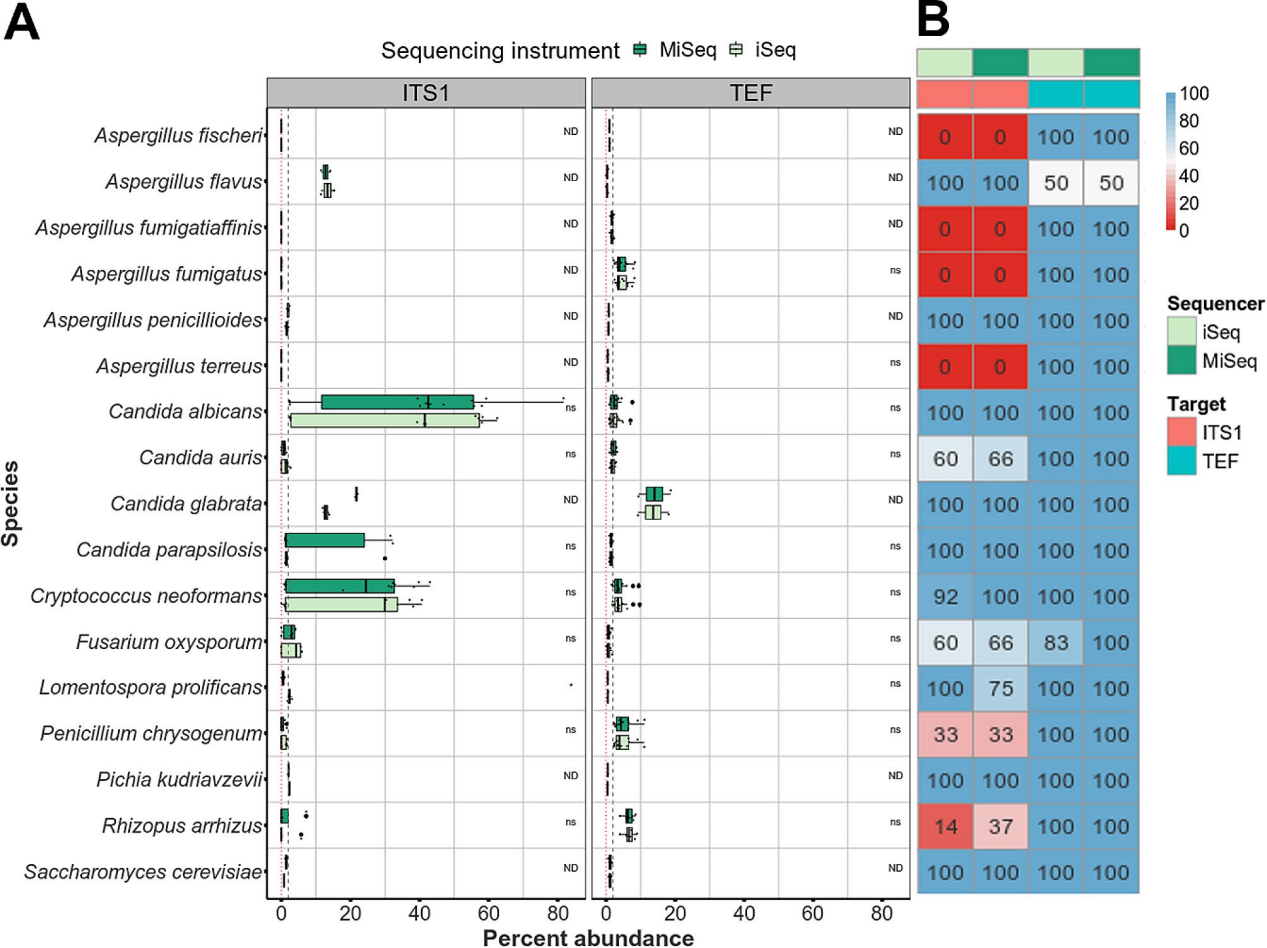
TEF metabarcoding assay outperforms ITS1 when detecting low level filamentous fungal species in mock communities. (**A**) Boxplot of percent abundance for species spiked at 2% within mock communities targeted by ITS1(left) or TEF (right). Expected percent abundance is indicated by dashed black line. The red dotted line indicates no identification. Percent abundance was significantly different between species for each target (*P* < 0.0001 by Kruskal-Wallis test). For each target, percent abundances are grouped by sequencing instrument. For those which were identified, excluding *Lomentospora prolificans* when targeted by ITS1, species level quantifications were not significantly different between instruments (ns, *P* < 0.05 by Wilcoxon rank sum test). Wilcoxon rank sum tests were not performed for species with less than 3 data points per human background status and are indicated with ‘ND’. (**B**) Heatmap representation of identification (ID) rates for species when spiked at 2% within mock communities. ITS1 failed to identify four filamentous fungi in any samples, and another five species had suboptimal ID rates. TEF identified all but one species in all samples tested. Sequencing instrument did not affect ID rates overall

Data from the 14 mock community samples (8 of which were sequenced in duplicate, denoted by x.2; input ratios provided in Table S4) were combined to assess the overall identification (ID) rate for species when spiked at 2% GEs (Fig. 4B). All samples were analysed on both iSeq and MiSeq platforms. Overall, ITS1 identified 13/17 species to some extent in the analysis. Five of the seven yeast species were identified in all samples in which they were present, regardless of instrument used. *C. neoformans* was detected in all but one sample (12/13; 92% detected) analysed on the MiSeq, however, *C. auris* had an ID rate of only 60 and 66% when sequenced on an iSeq and MiSeq, respectively. As expected, ITS1 performed particularly poorly in identifying filamentous fungal species, with only 2/10 species detected in all samples in which they were present. For *R. arrhizus, P. rubens* and *F. oxysporum*, ID rates ranged between 14 and 60% with iSeq and 33–66% with MiSeq (Fig. 4B). ITS1 failed to detect *A. fumigatus, A. fischeri, A. fumigatiaffinis* and *A. terreus* in any samples. In contrast, TEF successfully identified all 17 species tested, with 15 species identified in all samples in which they were present. *F. oxysporum* was detected in all samples when the MiSeq was used, but in only 83% of samples when the iSeq was used. *A. flavus* was identified in only half the samples tested (Fig. 4B).

To investigate the ability of each target to accurately quantify species present in samples at a low level (2% GEs) relative abundances were calculated from sequence data of all mock communities using read count as a proxy for DNA abundance (Fig. 4A). Median abundance for all low-level species was 1.1% with ITS1. However, per species median abundances ranged between 0 and 42.5%. As *P. rubens* and *R. arrhizus* were not identified in 67% and 75% of samples, respectively, the median abundances were zero for these species. Of the 13 species identified by ITS1, 7 had median abundances within 2-fold of the expected (0.4–4.2%). The remaining four species (*C. albicans, C. parapsilosis, Nakaseomyces (*formerly *Candida) glabrata* and *C. neoformans*) were particularly over-represented by ITS1, with median percent abundances ranging from 6 to 21-fold higher than expected (12.8–42.5%).

Of the 17 species identified by TEF, the majority [15] had abundances within 2-fold of expected values (0.3–4.3%). However, *N. glabrata* and *R. arrhizus* were over-represented, with median percent abundances ranging from 3 to 7-fold higher than expected (6.3–14.0%). For species with 3 or more data points per instrument and target, Wilcoxon rank sum tests were used to identify if sequencing instrument had a significant influence on species quantification. For most species, there was no significant difference between instruments, however, *L. prolificans* was under-represented by ITS1 on MiSeq, but not iSeq (0.5% with MiSeq, 2.3% with iSeq. *P* < 0.05).

### Human background has little impact on the overall identification rates by TEF

**Fig. 5:**
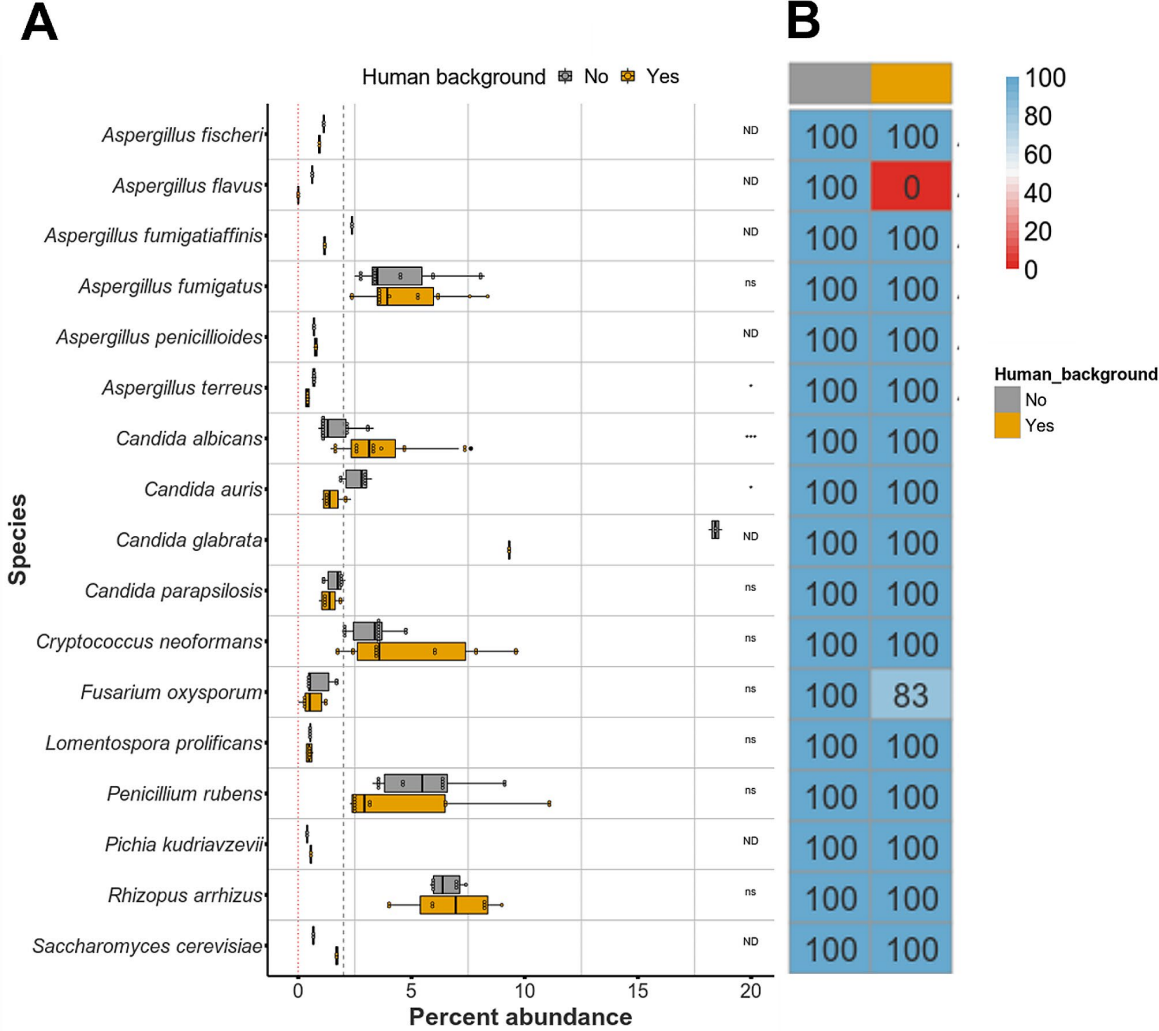
TEF species detection in mock fungal communities is not significantly hindered by human gDNA background. A. Boxplot of percent abundance for species spiked at 2% within mock communities targeted by TEF. Expected percent abundance is indicated by dashed black line. Dotted red line indicates no identification. Percent abundances are grouped by human DNA background status. Species level quantifications were significantly different when with and without human background for (**A**) *terreus, C. auris* and *C. albicans* (*; *P* < 0.05 or ***; *P* < 0.0001 by Wilcoxon rank sum test). For all other species which were identified in a sample, quantifications did not differ significantly (*P* < 0.05 by Wilcoxon rank sum test). Wilcoxon rank sum tests were not performed for species with less than 3 data points per human background status and are indicated with ‘ND’. (**B**) Heatmap representation of TEF identification (ID) rates for species when spiked at 2% within mock communities depending on human DNA background status. Human DNA resulted in no change for most species tested

To assess if the presence of human genomic DNA influenced the ability of TEF to identify fungal species at low abundance in a sample, mock communities containing the same relative proportions of fungal species plus a background of 10-fold (relative to total fungal copy number) haploid human genome equivalents were sequenced (Fig. 5). Although some differences in abundances were observed, there was no apparent pattern, as human background sometimes caused an increase or decrease in species abundance. For species with 3 or more data points per human background status, Wilcoxon rank sum tests were applied to identify if species quantification differed significantly. The addition of human genomic DNA background did not significantly influence the ability to identify most species when present at 2% in a mix (2% relative to other fungal DNA). However, in the presence of human background, abundance of *A. terreus* and *C. auris* reduced significantly (0.7 to 0.4 and 2.8 to 1.4%, respectively), although they remained detectable. In addition, human background resulted in a significant increase in *C. albicans* percent abundance (1.3 to 3.1%). Moreover, in the presence of a human background, *A. flavus* was not identified.

A similar experiment was performed to assess the impact of bacterial DNA background (˜ 2000-fold relative to total fungal copy number) on TEF fungal detection in mock communities and there was no difference in detection rates for the seven species tested with and without bacterial DNA background (Fig. S8).

### The Tef1 assay can detect as few as two genomes of *Aspergillus fumigatus* when spiked into clinical respiratory samples

**Fig. 6:**
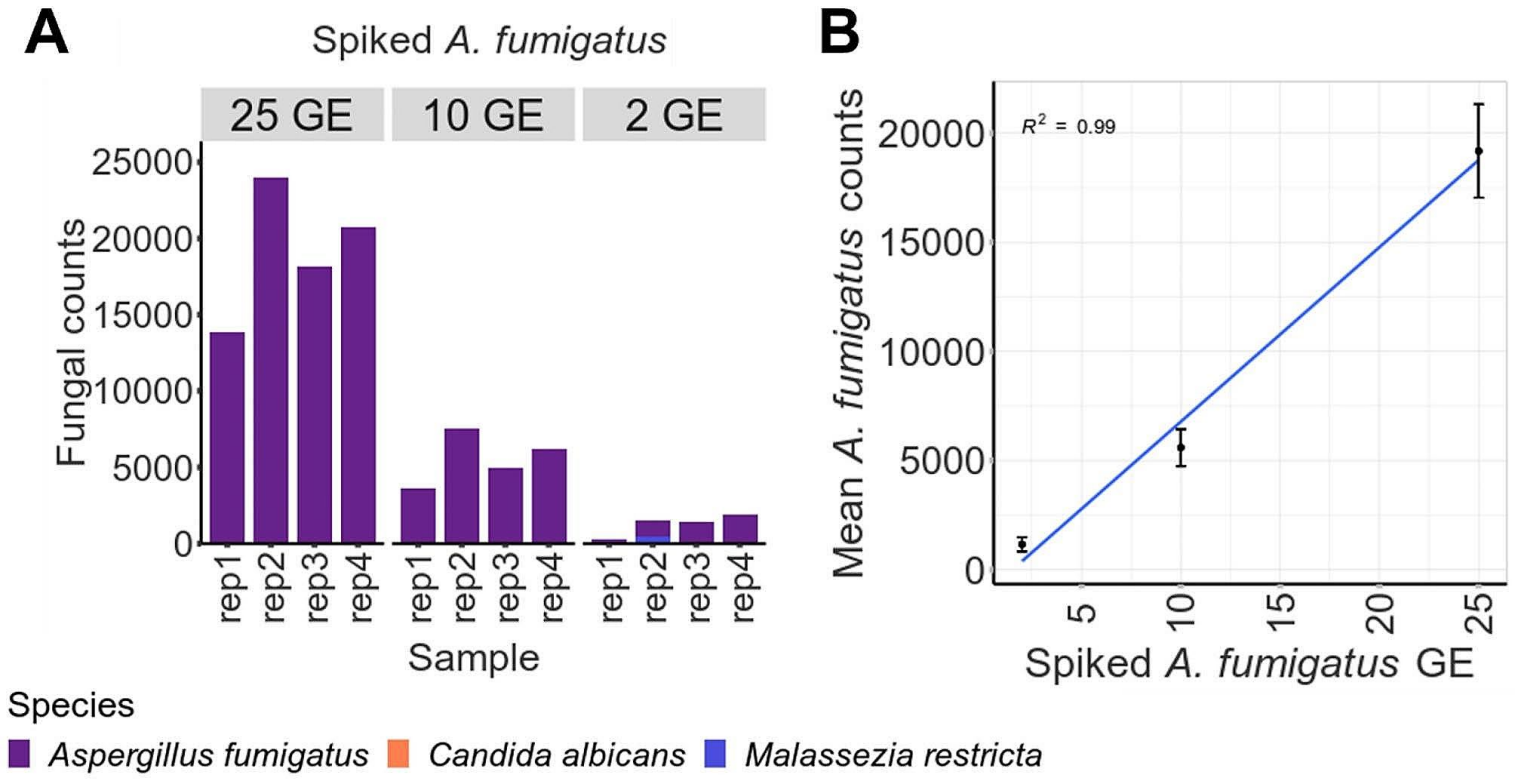
Assay evaluation using *A. fumigatus* spiked clinical respiratory samples. A sputum sample (previously negative for *Aspergillus* by ITS and TEF sequencing) spiked with varying quantities (2, 10 and 25 haploid genome equivalents/µl) of *A. fumigatus* gDNA. (**A**) Fungal read counts are shown for each sample in quadruplicate. Samples with less than 500 fungal reads were removed. No template control and unspiked sputum sample did not produce significant fungal read yields. (**B**) Mean *A. fumigatus* counts displayed a linear relationship with amount of spiked *A. fumigatus* (error bars indicated standard error). R2 value for linear relationship is indicated

The assay was tested against a clinical respiratory sample spiked with *A. fumigatus* gDNA. The assay was able to detect all quantities tested (25, 10 and 2 GE per reaction) in all four replicates analysed (Fig. 6A). The mean *A. fumigatus* read yield displayed a linear relationship with the spiked gDNA (Fig. 6B, R^2^ 0.99), suggesting the assay output has the potential to provide some degree of quantitation which could be informative for a clinical setting.

### Assay optimisation using clinical respiratory samples

The amplification method was optimised for use with clinical samples. For many fungal pathogens respiratory infection is the main clinical problem, therefore we used residual respiratory samples to optimise the TEF PCR. Using real and spiked clinical samples (*A. fumigatus* gDNA spiked at 0, 2 or 8 GE per reaction), a variety of PCR cycling parameters (annealing temperature and cycle number) and PCR additives (2% DMSO and 2 µg/µL BSA) were tested (Fig. S9A). PCR additives did not appear to improve the outcome, whereas increasing the annealing temperature resulted in the most *A. fumigatus* recovery from the samples. Finally, a comparison of PCR reagents, using Phusion master mix, Phusion master mix plus additives and KAPA master mix, was performed (alongside the optimised cycling parameters of 67 ℃ annealing temperature and 50 cycles) against *A. fumigatus* positive and negative clinical respiratory samples (Fig. S9B). The KAPA master mix resulted in optimal amplification of fungal DNA from samples, and thus was used for subsequent experiments.

### The optimised Tef1 assay can detect as few as two genomes of *Aspergillus fumigatus* in *Aspergillus*-positive clinical respiratory samples

The optimised method of the assay was applied to 10 clinical samples previously assessed by a clinically validated *Aspergillus fumigatus* qPCR test targeting the 18S rRNA gene. As samples had been in storage for a prolonged period (> 12 months), they were re-validated in-house to quantify the presence of *A. fumigatus* using a TaqMan qPCR assay targeting the ITS1 region [39]. Using a Ct cut-off of 40, 2 samples were negative, 3 were deemed positive at a level of < 1 GE and 5 at a level > 1 GE (Table 1). The TEF assay identified all 5 samples which were positive at > 1 GE per reaction but failed to identify samples positive at < 1 GE per reaction, resulting in a total of 5 true positives and 3 false negatives. The two negative samples were correctly identified therefore, no false positives were evident. The novel assay targets a single copy gene, hence a theoretical sensitivity limit of 1 genome per reaction is likely. Therefore, these results suggest the assay is working close to its theoretical capabilities.

**Table 1:** Assay evaluation using clinical respiratory samples. Ten clinical respiratory samples were assessed using TaqMan qPCR to determine *Aspergillus fumigatus* positivity. Two samples were negative for *A. fumigatus* (labelled QN). Three samples contained *A. fumigatus* DNA at a positivity level below 1 GE per reaction, and 5 samples at a positivity level above 1 GE per reaction (shown in bold). All samples above the sequencing assay’s theoretical detection limit (1 GE/reaction) (n=5) were identified as *A. fumigatus* positive by TEF sequencing

Sample	Mean Ct	Mean GE/ reaction	qPCR result	Sequencing result
QP1	34.49	2.1	+	+
QP2	33.89	3.3	+	+
QP3	36.37	0.5	+	-
QP4	36.60	0.4	+	-
**QP5**	**32.13**	**12.6**	**+**	**+**
**QP6**	**29.99**	**65.2**	**+**	**+**
QP7	37.11	0.3	+	-
**QP8**	**28.08**	**283.1**	**+**	**+**
QN1	48.87	0.0	-	-
QN2	n/a	n/a	-	-

GE; haploid genome equivalents

Ct - threshold cycle

### Spike-in controls enable semi-quantitation of *Aspergillus fumigatus* load in clinical respiratory samples using sequencing data

**Fig. 7:**
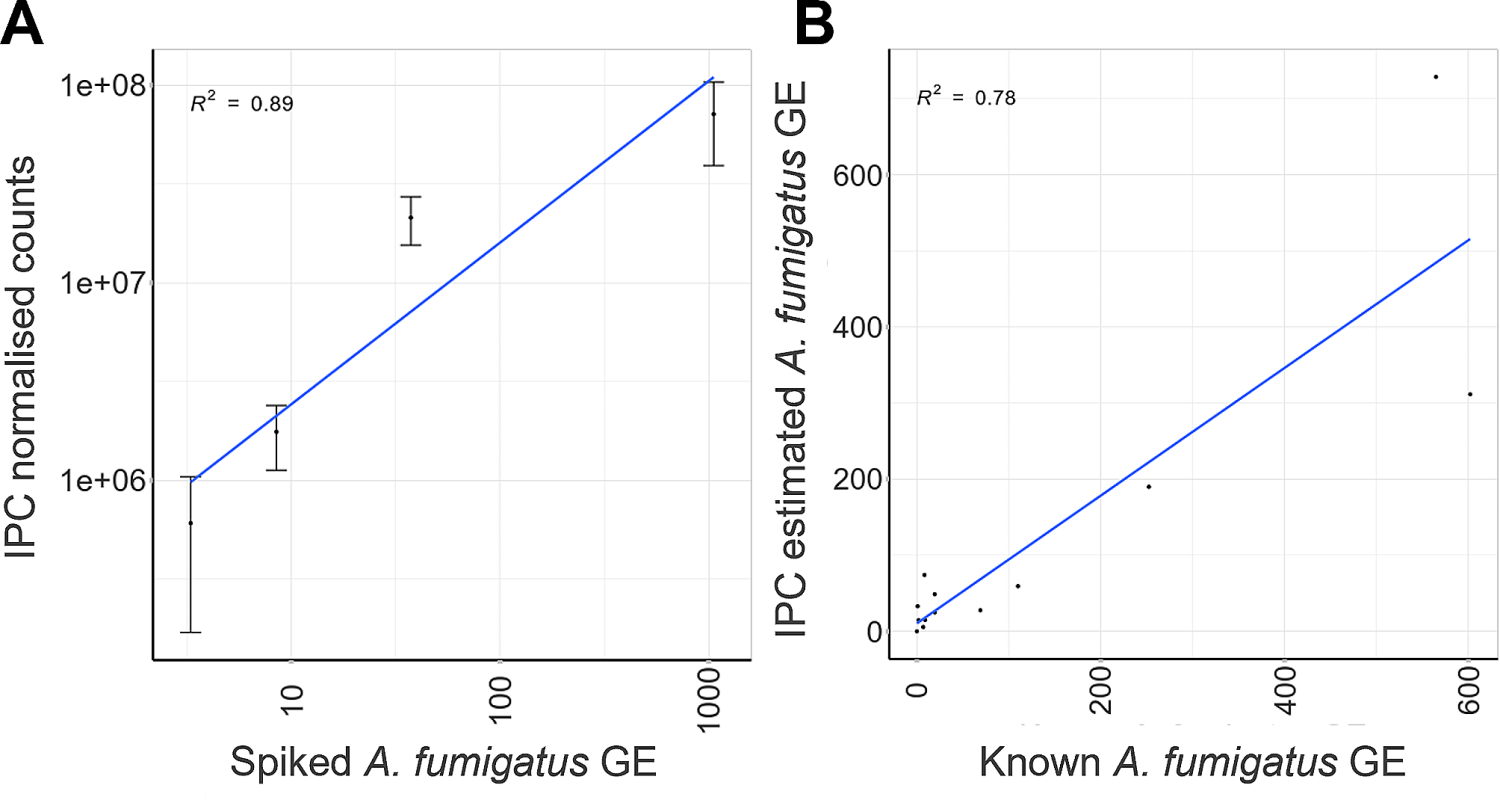
Absolute quantification of *Aspergillus fumigatus* using internal plasmid control PC1. A. Clinical respiratory samples were spiked with four different amounts of *A. fumigatus* (*A. f*) genome equivalents (GE) to generate a standard curve ranging from ˜ 3 to 1000 GE per PCR. Read counts from the internal plasmid control (PC) were used to normalize (**A**) *f* counts and a positive linear relationship (R2 = 0.89) with spiked GE was observed. (**B**) Using the linear model generated by the standard curve, *A. f* GE were estimated in 12 clinical respiratory samples known to be positive for *A. f* (plus one negative control sample containing no *A. f*). Estimated *A. f* GE for test samples were calibrated using the estimated *A. f* GE value of the negative control sample. The estimated *A. f* GE values displayed strong correlation (Spearman’s rank-order correlation; *r* [11] 0.78, *p* < 0.01) with known GE (calculated by rRNA qPCR)

We next sought to determine whether the TEF assay could improve quantitation. Standard microbiome methods produce compositional data, which only provide relative abundance information [48]. Some studies have achieved absolute abundances using complimentary techniques such as droplet digital PCR and flow cytometry alongside microbiome analysis [48, 49]. Our aim was to develop a system where abundance measured by read count depth had a clinically useful relationship with pathogen level in the sample for important disease-causing species without adding the expense and time of additional assays. To enable this, we designed and implemented internal plasmid controls (IPCs) to act as spike-in DNA. Synthetic DNA spikes for quantitation have been demonstrated for bacterial and eukaryotic microbiome analyses of environmental samples [50] but is yet to be adopted broadly. As a proof of principle, we first assessed the accuracy of generating a standard curve by spiking qPCR negative clinical respiratory samples with known amounts of *A. fumigatus* gDNA covering three orders of magnitude (˜ 3 to ˜ 1000 GE/reaction) and three different IPCs (IPC5, IPC4 and IPC1 at 10, 30 and 90 copies per µL, respectively). The level of *A. fumigatus* gDNA in these spiked standard curve samples was validated using TaqMan qPCR. Upon sequencing, *A. fumigatus* read counts were normalised using the relative amount of reads for each IPC. For all the IPCs tested, the normalised data had high correlation (R^2^ > 0.8) with the known *A. fumigatus* GE (Fig. S10A-C, IPC1 data shown in Fig. 7A). We then assessed the use of such standard curves as a reference for quantifying A. *fumigatus* in qPCR positive clinical respiratory samples spiked with IPCs. The amount of *A. fumigatus* in the positive clinical respiratory samples was quantified using a linear model of the standard curve data. A negative control sample (respiratory sample negative for *A. fumigatus* by qPCR) was used to calibrate the GE estimations generated. With two IPCs, the estimated GE values had poor correlation with known GE (calculated by qPCR): R^2^ 0.44 and 0.07 for IPC4 and IPC5, respectively (Fig. S10G-I). However, calculations using IPC1 provided good correlation with known GE values (R^2^ 0.78) (Fig. 7B). Therefore, we have demonstrated that an IPC can be spiked into the assay to provide results which give an indication of the fungal load in a patient sample.

## Discussion

This study designed a novel fungal metabarcoding amplicon targeting a single copy gene, TEF. PCR analyses *in silico* suggest broad fungal coverage and little variation in amplicon size. PCR experiments demonstrated TEF to provide more reliable detection and potentially, quantification of fungal gDNA compared to ITS1. The variability in species detection by ITS1 could be due to primer mismatches (as is likely the case for the reduced efficiency observed with *L. prolificans*) or due to variation in rRNA copy number between fungal isolates.

Problems are often encountered when relying on public databases as they can be error-prone and misleading when trying to assign taxa to sequence data [51]. Although curated fungal sequence databases exist, they generally focus on ITS sequence data [18]. Use of TEF as a secondary barcode has gained traction, as evidenced by the recent emergence of the ISHAM TEF sequence database, and thus available reference data is continually increasing for this target. However, as the ISHAM TEF database remains small (< 150 species), we developed and curated a fungal database covering 6008 species to facilitate metabarcoding analysis. Investigating these available sequences, the novel amplicon was demonstrated to have good speciation capabilities against a set of 30 clinically relevant fungal pathogens. However, one of the two *S. dehoogi* sequences is identical to that of *Scedosporium boydii*; whether this interspecies sequence overlap is a true reflection of sequence similarity or database error is unclear, and more data is needed to resolve this. In contrast, unlike ITS1, TEF was able to speciate *Fusarium oxysporum* and *Fusarium acutatum*. When comparing the speciation capabilities within *Aspergillus*, both targets were unable to distinguish between some members of the *A. fumigatus* SC (e.g. *A. lentulus*, *A. novofumigatus* and *A. viridinutans*). TEF displayed slightly improved speciation here, as it was able to differentiate *A. fischeri* from these species. As previously shown [47], ITS1 had poor performance within the *A. niger* SC and was unable to differentiate between the seven species assessed here. TEF was able to delineate the species within this complex. TEF and ITS1 were both unable to differentiate *A. flavus* from the closely related and less pathogenic species *A. oryzae.* The available ITS1 sequences for the *A. flavus* SC also illustrated how considerable the intraspecies variation can be with this target, which is well described for ITS targets overall [18, 52]. Less intraspecies variation was found with TEF, as previously described for TEF compared to ITS targets [18]. Therefore, the speciation ability of the TEF amplicon was similar but not entirely identical to that of ITS1 and has less apparent intraspecies variation. Nevertheless, neither target is able to fully speciate members of species-rich genera such as *Aspergillus*, and hence why the use of two barcoding targets (e.g. a combination of ITS and *TEF1a* targets) has been suggested where full species delineation is required [53]. To this end, there is future scope for the TEF assay to be combined with another amplicon target to potentially establish a dual-metabarcoding assay with improved species-level identification where necessary.

Most environmental or clinical samples contain mixtures of fungi with different abundances. To test the ability of ITS1 and TEF amplicons to speciate DNA in mixed samples, mock communities were sequenced. ITS1 significantly under-represented or did not detect filamentous fungi, such as *A. fumigatus*, when present in mixtures containing even low levels of yeast species. In addition, when sequencing filamentous fungi with ITS1 in isolation, lower read numbers were recovered in comparison to yeast. The TEF amplicon was able to accurately identify the compositions of all mock communities tested. The presence of human DNA background mostly had little impact on the species identification rates with TEF. However, 2% *A. flavus* was no longer identified when in the presence of 10-fold human DNA background. The reason for loss of detection of this species in particular is unknown. One explanation for such a loss would be that the human DNA causes competitive inhibition due to sequence similarity to primer regions in the human eEF1A gene. However, the similarity of the *A. flavus* TEF primer region sequences to human eEF1A is less than for *A. terreus*, a species which was also tested here but remained detectable the presence of human background. Therefore, the loss of *A. flavus* detection is a finding which could be further validated and explored in a larger sample set.

Optimisation of the TEF assay was performed to improve performance with clinical respiratory samples. Increasing the annealing temperature improved recovery of *A. fumgiatus* from clinical respiratory samples. This finding may have been due to improved primer binding specificity; however, it could possibly have reduced performance for other clinically relevant fungal pathogens which were not tested in this experiment, and this could be investigated further in future studies. TEF amplicon sequencing was able to detect as few as two *A. fumigatus* genomes from clinical respiratory samples, suggesting the assay is performing close to the theoretical detection limit of one genome. This intrinsic limit, due to the single copy nature of the target, is one limitation of the assay. Although single copy targets allow more accurate quantification in comparison to a variable copy target, sensitivity can never be comparable to a multicopy target such as rRNA regions [52, 54].

Clinical diagnostic tests which provide quantitative results are helpful in determining pathogen burden in a patient sample. To this end, we described a proof-of-principle for the use of spike-in control DNA to allow quantitation of fungus in respiratory samples without the need for additional tests. Although estimated fungal levels correlated well with qPCR-quantified burden, estimated values varied up to 2-fold from the reference for samples with particularly high fungal load (> 300 GE). Considering this variation, the method may be suitable for stratifying samples into low, medium, or high burden categories. Nevertheless, this provides semi-quantitative data, which contrasts with established mycobiome sequencing methods which provide no estimate of absolute DNA abundance [55]. One limitation of this comparison is the use of a multicopy qPCR target to validate the presence of *A. fumigatus* in the clinical samples, as the previously discussed issues regarding copy number variation may have interfered with accurate quantification of burden.

The high-throughput nature of NGS is attractive for diagnostic laboratories with high sample turnover because it allows for detection of a wide range of species in multiple samples in a single assay. The assay developed here also uniquely barcodes samples in the first PCR step, allowing sample pooling immediately after this, reducing hands-on time and reagent needs. This method offers an advantage over a standard Illumina Nextera metabarcoding procedure, which requires samples to be processed individually until much later in the protocol. We also demonstrated that the assay is compatible and gives consistent results with cost-effective sequencing options, using either the low cost iSeq 100 sequencer or a MiSeq with the low-cost micro reagent kit. Estimated reagent costs per sample are ˜ 13.50 EURO, calculated based on an iSeq sequencing run analysing 48 samples and including controls. Therefore, this novel assay is a flexible and affordable approach which can survey fungal populations within multiple samples simultaneously with reduced processing during library preparation. Moreover, as the assay does not aim to perform in-depth community-based analysis or comparison, downstream bioinformatic processing is simpler to run than traditional microbiome research methods, and analysis pipelines provide results for each sample as a straightforward, sorted list of species counts per sample and can also integrate quantitation when spike-in DNA is utilised. In addition, we have generated resources which make the output compatible with the established microbiome analysis package, Phyloseq, hence providing the option to produce simple visualisation of data if desired [33].

As the patient sample set used here is of limited size, further validation with a larger cohort is necessary. Before a metabarcoding assay can be implemented in a clinical setting, there needs to consideration regarding the parameters or implications of a result [56]. When the sample contains a high level of a common fungal pathogen, the clinical implication may be clear. However, the finding of a variety of less common fungal species in a sample is not immediately informative in a clinical setting. Therefore, the potentially complex results of such a test would benefit from additional information and guidelines on interpretation. Evidence for such guidelines could be generated by a large-scale validation study of the assay in clinical practice.

## Conclusion

A novel fungal metabarcoding assay targeting a single-copy gene was developed and evaluated. The method was designed to be more time- and cost-effective than standard metabarcoding approaches and its performance successfully demonstrated using the affordable Illumina iSeq100 sequencer that requires low operator input. The TEF assay outperformed the ITS1 region for identification of filamentous fungi in mixed, mock communities. The metabarcoding assay was also shown to identify as few as 2 genomes of *A. fumigatus* from clinical respiratory samples and semi-quantitative results were achieved from sequencing data by including spike-in control DNA. The assay described here is a promising diagnostic candidate that could provide pan-fungal identification and speciation from clinical sample DNA extracts in an affordable manner.

### Electronic supplementary material

Below is the link to the electronic supplementary material.

Additional file 2: Supplementary_figures.pdf

Supplementary Material 2

Supplementary Material 3

Supplementary Material 4

Additional file 1: Supplementary_tables.xlsx

Supplementary Material 6

## Data Availability

Raw sequence data has been deposited at the NCBI sequence read archive (SRA) under accession number PRJNA980461. Code used for analysis is available at https://github.com/Danweaver1/Amplicon-sequencing-diagnostics.

## Abbreviations

bp: Basepairs
Ct: Threshold cycle
gDNA: Genomic DNA
GE: Haploid genome equivalents
ID: Identification
IPC: Internal plasmid control
ITS: Internal transcribed spacer
LOD: Limit of detection
NGS: Next generation sequencing
SC: Species complex
TEF: Translation elongation factor
