# Supplementary material for: Development of a novel mycobiome diagnostic for fungal infection
Source: BMC Microbiol. 2024 Feb 19;24:63. doi: 10.1186/s12866-024-03197-5 (PMC10875777; doi:10.1186/s12866-024-03197-5)
Supplement: Supplementary file 1 — Additional file 2: Supplementary_figures.pdf [file 12866_2024_3197_MOESM1_ESM.pdf]

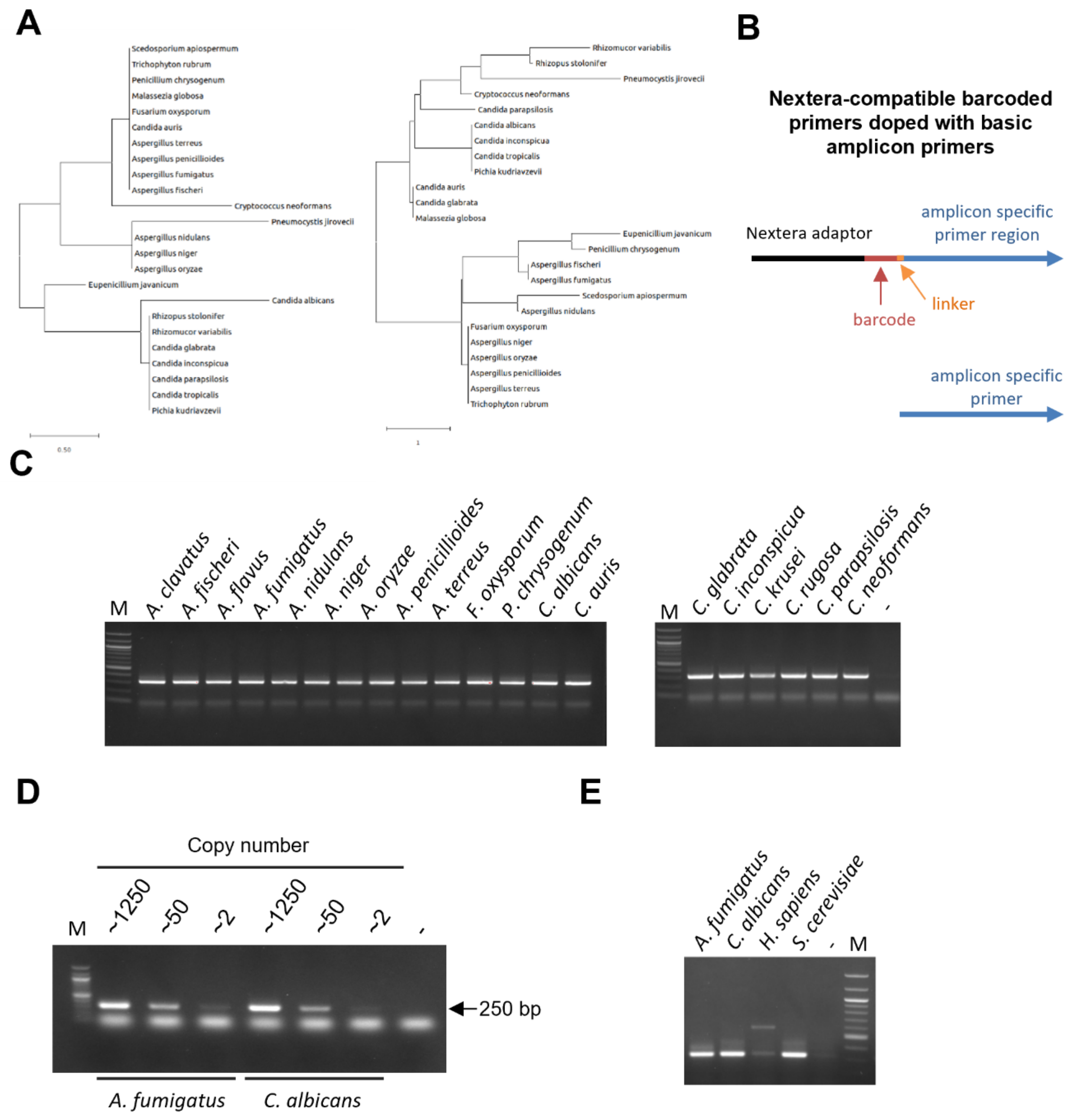

Figure S1. TEF PCR assay. A. Schematic of TEF NGS-compatible PCR assay oligo design. B. Primer sequence groupings of fungal species used for designing TEF1α multiplex PCR assay. The evolutionary history was inferred using the Neighbor-Joining method [1]. The evolutionary distances were computed using the number of differences method [2] and are in the units of the number of base differences per sequence. Evolutionary analyses were conducted in MEGA X [3]. Trees represent forward (A) and reverse (B) primer sequences. The two major branches of each tree represent coverage of each primer. C. PCR validation of fungal species coverage of TEF assay. Reactions included 5 nanogram of each species. Samples were analysed over two agarose gels, as shown. Each gel has an associated DNA marker. D. Sensitivity of TEF assay. PCR reactions included ~1250, 50 or 2 haploid genome equivalents of *A. fumigatus* or *C. albicans* and a template control. E. Comparison of TEF assay with fungal and human genomic DNA. Reactions included 5 ng of each species. All agarose gels include a 100bp DNA Marker (NEB) for size reference. Source images for the cropped gel images include here are provided in figure S9.

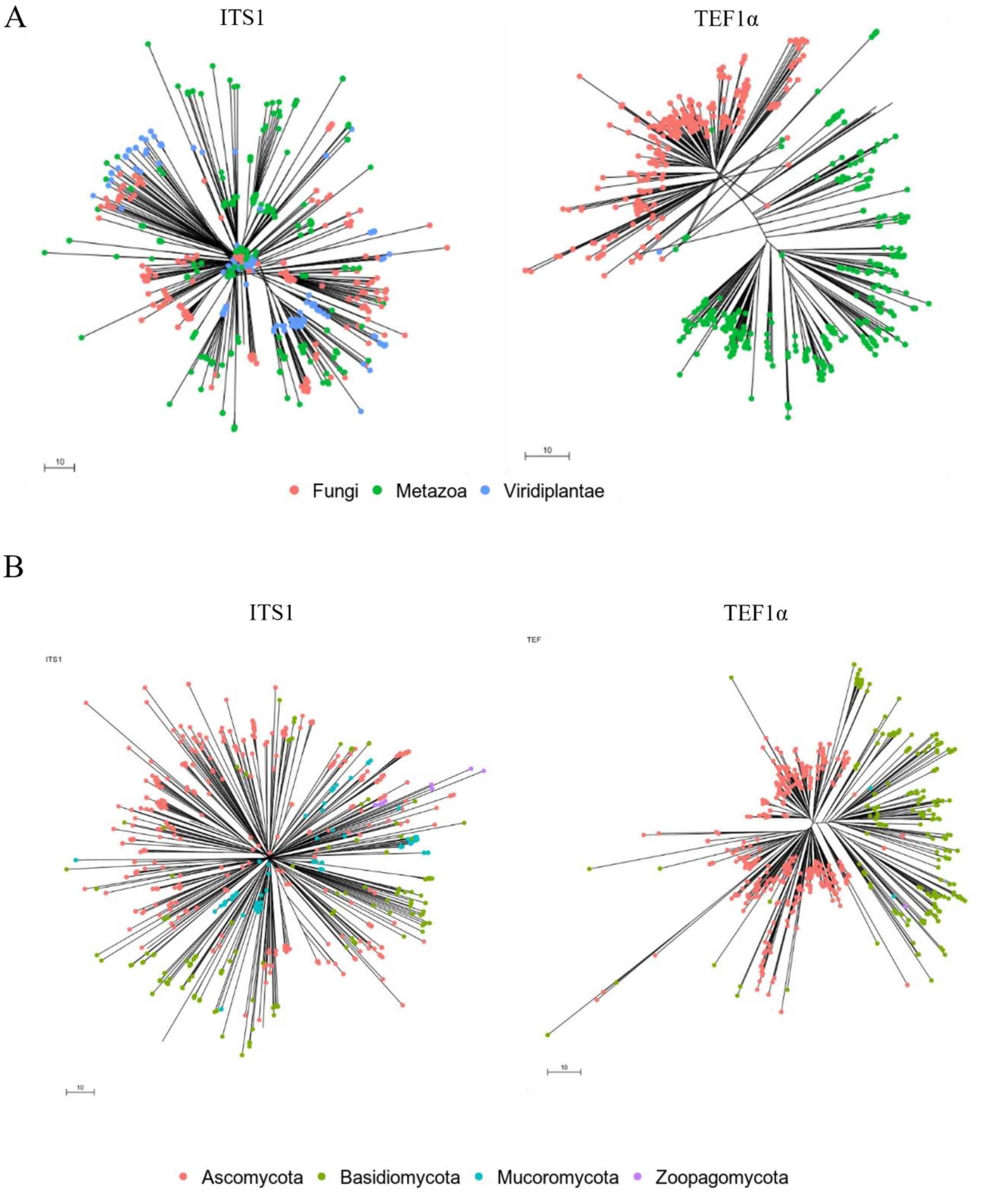

Figure S2. ***in silico*** **PCR performance of ITS1 and TEF targets.** PrimerTree was used to search primers against NCBI non redundant nucleotide database. Unrooted tree indicates taxonomic kingdom of identified sequences for ITS1 (left) and TEF1α. B. PrimerTree searches were repeated against fungal sequences only. Radial trees indicate the fungal phyla of hits for ITS1 (top) and TEF1α.

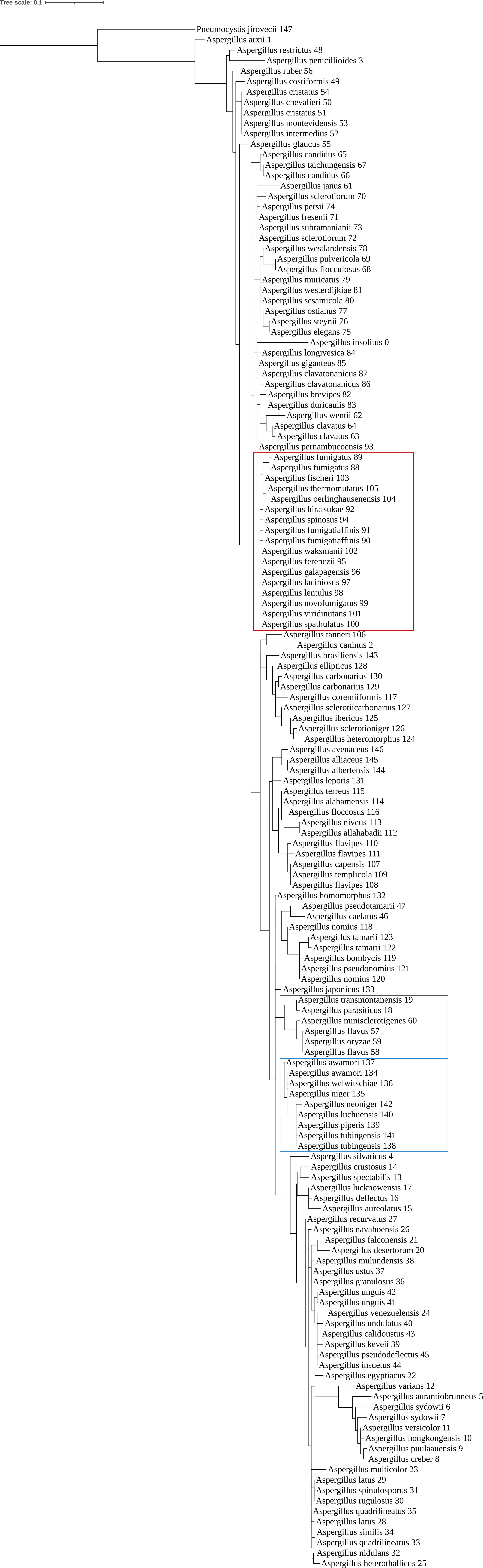
Tree scale: 0.1
Pneumocystis jirovecii 147
Aspergillus arxii 1
Aspergillus restrictus 48
Aspergillus penicillioides 3
Aspergillus ruber 56
Aspergillus costiformis 49
Aspergillus cristatus 54
Aspergillus chevalieri 50
Aspergillus cristatus 51
Aspergillus montevidensis 53
Aspergillus intermedius 52
Aspergillus glaucus 55
Aspergillus candidus 65
Aspergillus taichungensis 67
Aspergillus candidus 66
Aspergillus janus 61
Aspergillus sclerotiorum 70
Aspergillus persii 74
Aspergillus fresenii 71
Aspergillus subramanianii 73
Aspergillus sclerotiorum 72
Aspergillus westlandensis 78
Aspergillus pulvericola 69
Aspergillus flocculosus 68
Aspergillus muricatus 79
Aspergillus westerdijkiae 81
Aspergillus sesamicola 80
Aspergillus ostianus 77
Aspergillus steynii 76
Aspergillus elegans 75
Aspergillus insolitus 0
Aspergillus longivesica 84
Aspergillus giganteus 85
Aspergillus clavatonanicus 87
Aspergillus clavatonanicus 86
Aspergillus brevipes 82
Aspergillus duricaulis 83
Aspergillus wentii 62
Aspergillus clavatus 64
Aspergillus clavatus 63
Aspergillus pernambucoensis 93
Aspergillus fumigatus 89
Aspergillus fumigatus 88
Aspergillus fischeri 103
Aspergillus thermomutatus 105
Aspergillus oerlinghausenensis 104
Aspergillus hiratsukae 92
Aspergillus spinosus 94
Aspergillus fumigatiaffinis 91
Aspergillus fumigatiaffinis 90
Aspergillus waksmanii 102
Aspergillus ferenczii 95
Aspergillus galapagensis 96
Aspergillus laciniosus 97
Aspergillus lentulus 98
Aspergillus novofumigatus 99
Aspergillus viridinutans 101
Aspergillus spathulatus 100
Aspergillus tanneri 106
Aspergillus caninus 2
Aspergillus brasiliensis 143
Aspergillus ellipticus 128
Aspergillus carbonarius 130
Aspergillus carbonarius 129
Aspergillus coremiiformis 117
Aspergillus sclerotiicarbonarius 127
Aspergillus ibericus 125
Aspergillus sclerotioniger 126
Aspergillus heteromorphus 124
Aspergillus avenaceus 146
Aspergillus alliaceus 145
Aspergillus albertensis 144
Aspergillus leporis 131
Aspergillus terreus 115
Aspergillus alabamensis 114
Aspergillus floccosus 116
Aspergillus niveus 113
Aspergillus allahabadii 112
Aspergillus flavipes 110
Aspergillus flavipes 111
Aspergillus capensis 107
Aspergillus templicola 109
Aspergillus flavipes 108
Aspergillus homomorphus 132
Aspergillus pseudotamarii 47
Aspergillus caelatus 46
Aspergillus nomius 118
Aspergillus tamarii 123
Aspergillus tamarii 122
Aspergillus bombycis 119
Aspergillus pseudonomius 121
Aspergillus nomius 120
Aspergillus japonicus 133
Aspergillus transmontanensis 19
Aspergillus parasiticus 18
Aspergillus minisclerotigenes 60
Aspergillus flavus 57
Aspergillus oryzae 59
Aspergillus flavus 58
Aspergillus awamori 137
Aspergillus awamori 134
Aspergillus welwitschiae 136
Aspergillus niger 135
Aspergillus neoniger 142
Aspergillus luchuensis 140
Aspergillus piperis 139
Aspergillus tubingensis 141
Aspergillus tubingensis 138
Aspergillus silvaticus 4
Aspergillus crustosus 14
Aspergillus spectabilis 13
Aspergillus lucknowensis 17
Aspergillus deflectus 16
Aspergillus aureolatus 15
Aspergillus recurvatus 27
Aspergillus navahoensis 26
Aspergillus falconensis 21
Aspergillus desertorum 20
Aspergillus mulundensis 38
Aspergillus ustus 37
Aspergillus granulosus 36
Aspergillus unguis 42
Aspergillus unguis 41
Aspergillus venezuelensis 24
Aspergillus undulatus 40
Aspergillus calidoustus 43
Aspergillus keveii 39
Aspergillus pseudodeflectus 45
Aspergillus insuetus 44
Aspergillus egyptiacus 22
Aspergillus varians 12
Aspergillus aurantiobrunneus 5
Aspergillus sydowii 6
Aspergillus sydowii 7
Aspergillus versicolor 11
Aspergillus hongkongensis 10
Aspergillus puulaauensis 9
Aspergillus creber 8
Aspergillus multicolor 23
Aspergillus latus 29
Aspergillus spinulosporus 31
Aspergillus rugulosus 30
Aspergillus quadrilineatus 35
Aspergillus latus 28
Aspergillus similis 34
Aspergillus quadrilineatus 33
Aspergillus nidulans 32
Aspergillus heterothallicus 25

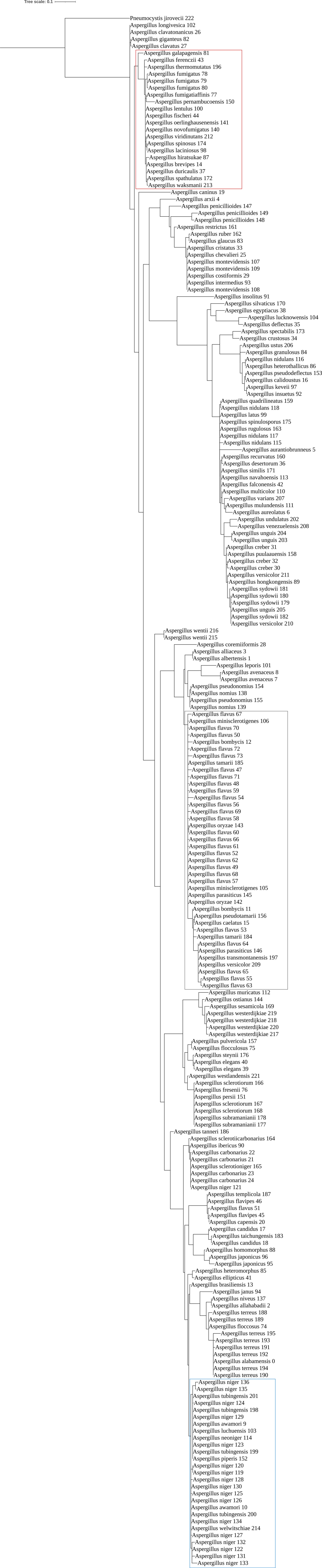

Tree scale: 0.1
Pneumocystis jirovecii 222
Aspergillus longivesica 102
Aspergillus clavatonanicus 26
Aspergillus giganteus 82
Aspergillus clavatus 27
Aspergillus galapagensis 81
Aspergillus ferenczii 43
Aspergillus thermomutatus 196
Aspergillus fumigatus 78
Aspergillus fumigatus 79
Aspergillus fumigatus 80
Aspergillus fumigatiaffinis 77
Aspergillus pernambucoensis 150
Aspergillus lentulus 100
Aspergillus fischeri 44
Aspergillus oerlinghausenensis 141
Aspergillus novofumigatus 140
Aspergillus viridinutans 212
Aspergillus spinosus 174
Aspergillus laciniosus 98
Aspergillus hiratsukae 87
Aspergillus brevipes 14
Aspergillus duricaulis 37
Aspergillus spathulatus 172
Aspergillus waksmanii 213
Aspergillus caninus 19
Aspergillus arxii 4
Aspergillus penicillioides 147
Aspergillus penicillioides 149
Aspergillus penicillioides 148
Aspergillus restrictus 161
Aspergillus ruber 162
Aspergillus glaucus 83
Aspergillus cristatus 33
Aspergillus chevalieri 25
Aspergillus montevidensis 107
Aspergillus montevidensis 109
Aspergillus costiformis 29
Aspergillus intermedius 93
Aspergillus montevidensis 108
Aspergillus insolitus 91
Aspergillus silvaticus 170
Aspergillus egyptiacus 38
Aspergillus lucknowensis 104
Aspergillus deflectus 35
Aspergillus spectabilis 173
Aspergillus crustosus 34
Aspergillus ustus 206
Aspergillus granulosus 84
Aspergillus nidulans 116
Aspergillus heterothallicus 86
Aspergillus pseudodeflectus 153
Aspergillus calidoustus 16
Aspergillus keveii 97
Aspergillus insuetus 92
Aspergillus quadrilineatus 159
Aspergillus nidulans 118
Aspergillus latus 99
Aspergillus spinulosporus 175
Aspergillus rugulosus 163
Aspergillus nidulans 117
Aspergillus nidulans 115
Aspergillus aurantiobrunneus 5
Aspergillus recurvatus 160
Aspergillus desertorum 36
Aspergillus similis 171
Aspergillus navahoensis 113
Aspergillus falconensis 42
Aspergillus multicolor 110
Aspergillus varians 207
Aspergillus mulundensis 111
Aspergillus aureolatus 6
Aspergillus undulatus 202
Aspergillus venezuelensis 208
Aspergillus unguis 204
Aspergillus unguis 203
Aspergillus creber 31
Aspergillus puulaauensis 158
Aspergillus creber 32
Aspergillus creber 30
Aspergillus versicolor 211
Aspergillus hongkongensis 89
Aspergillus sydowii 181
Aspergillus sydowii 180
Aspergillus sydowii 179
Aspergillus unguis 205
Aspergillus sydowii 182
Aspergillus versicolor 210
Aspergillus wentii 216
Aspergillus wentii 215
Aspergillus coremiiformis 28
Aspergillus alliaceus 3
Aspergillus albertensis 1
Aspergillus leporis 101
Aspergillus avenaceus 8
Aspergillus avenaceus 7
Aspergillus pseudonomius 154
Aspergillus nomius 138
Aspergillus pseudonomius 155
Aspergillus nomius 139
Aspergillus flavus 67
Aspergillus minisclerotigenes 106
Aspergillus flavus 70
Aspergillus flavus 50
Aspergillus bombycis 12
Aspergillus flavus 72
Aspergillus flavus 73
Aspergillus tamarii 185
Aspergillus flavus 47
Aspergillus flavus 71
Aspergillus flavus 48
Aspergillus flavus 59
Aspergillus flavus 54
Aspergillus flavus 56
Aspergillus flavus 69
Aspergillus flavus 58
Aspergillus oryzae 143
Aspergillus flavus 60
Aspergillus flavus 66
Aspergillus flavus 61
Aspergillus flavus 52
Aspergillus flavus 62
Aspergillus flavus 49
Aspergillus flavus 68
Aspergillus flavus 57
Aspergillus minisclerotigenes 105
Aspergillus parasiticus 145
Aspergillus oryzae 142
Aspergillus bombycis 11
Aspergillus pseudotamarii 156
Aspergillus caelatus 15
Aspergillus flavus 53
Aspergillus tamarii 184
Aspergillus flavus 64
Aspergillus parasiticus 146
Aspergillus transmontanensis 197
Aspergillus versicolor 209
Aspergillus flavus 65
Aspergillus flavus 55
Aspergillus flavus 63
Aspergillus muricatus 112
Aspergillus ostianus 144
Aspergillus sesamicola 169
Aspergillus westerdijkiae 219
Aspergillus westerdijkiae 218
Aspergillus westerdijkiae 220
Aspergillus westerdijkiae 217
Aspergillus pulvericola 157
Aspergillus flocculosus 75
Aspergillus steynii 176
Aspergillus elegans 40
Aspergillus elegans 39
Aspergillus westlandensis 221
Aspergillus sclerotiorum 166
Aspergillus fresenii 76
Aspergillus persii 151
Aspergillus sclerotiorum 167
Aspergillus sclerotiorum 168
Aspergillus subramanianii 178
Aspergillus subramanianii 177
Aspergillus tanneri 186
Aspergillus sclerotiicarbonarius 164
Aspergillus ibericus 90
Aspergillus carbonarius 22
Aspergillus carbonarius 21
Aspergillus sclerotioniger 165
Aspergillus carbonarius 23
Aspergillus carbonarius 24
Aspergillus niger 121
Aspergillus templicola 187
Aspergillus flavipes 46
Aspergillus flavus 51
Aspergillus flavipes 45
Aspergillus capensis 20
Aspergillus candidus 17
Aspergillus taichungensis 183
Aspergillus candidus 18
Aspergillus homomorphus 88
Aspergillus japonicus 96
Aspergillus japonicus 95
Aspergillus heteromorphus 85
Aspergillus ellipticus 41
Aspergillus brasiliensis 13
Aspergillus janus 94
Aspergillus niveus 137
Aspergillus allahabadii 2
Aspergillus terreus 188
Aspergillus terreus 189
Aspergillus floccosus 74
Aspergillus terreus 195
Aspergillus terreus 193
Aspergillus terreus 191
Aspergillus terreus 192
Aspergillus alabamensis 0
Aspergillus terreus 194
Aspergillus terreus 190
Aspergillus niger 136
Aspergillus niger 135
Aspergillus tubingensis 201
Aspergillus niger 124
Aspergillus tubingensis 198
Aspergillus niger 129
Aspergillus awamori 9
Aspergillus luchuensis 103
Aspergillus neoniger 114
Aspergillus niger 123
Aspergillus tubingensis 199
Aspergillus piperis 152
Aspergillus niger 120
Aspergillus niger 119
Aspergillus niger 128
Aspergillus niger 130
Aspergillus niger 125
Aspergillus niger 126
Aspergillus awamori 10
Aspergillus tubingensis 200
Aspergillus niger 134
Aspergillus welwitschiae 214
Aspergillus niger 127
Aspergillus niger 132
Aspergillus niger 122
Aspergillus niger 131
Aspergillus niger 133

Figure S3. *Aspergillus* speciation of ITS1 and TEF1α amplicons. TEF1α (top) and ITS1 (bottom) amplicon sequences (without primer regions) were obtained for 131 *Aspergillus* species. Alignments were performed using Clustal. Phylogenetic trees were generated using the Maximum Likelihood method and Tamura-Nei model, with 500 bootstraps. Alignments and trees were created in MEGA X. Tree images were generated using iTOL. Coloured boxes indicated examples of species complexes (*Aspergillus fumigatus* in red, *Aspergillus flavus* in grey and *Aspergillus niger* in blue).

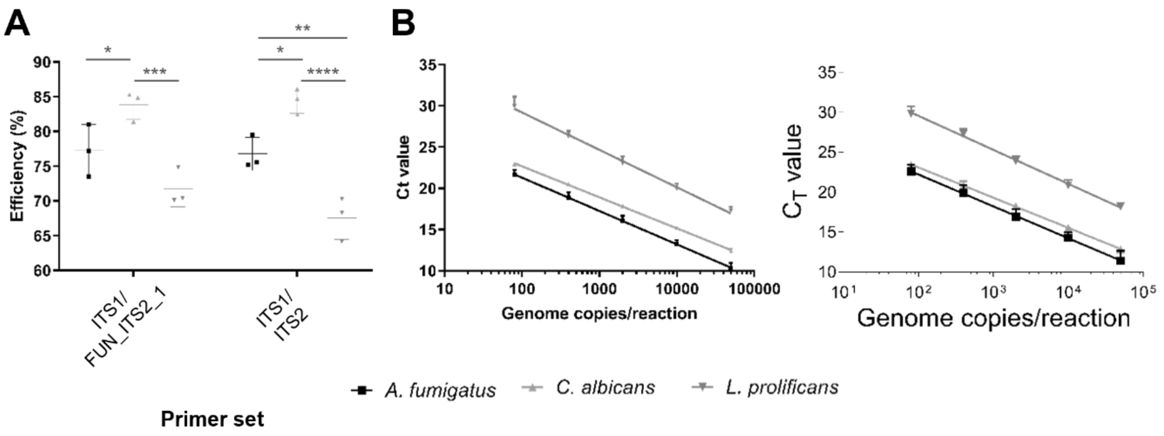

Figure S4. Efficiency and relative quantitation of human pathogenic fungal species with ITS1 primer sets. A. Quantitative PCR efficiency of primer sets ITS1/2 and ITS1/FUN_ITS2_1. B. Relative quantitation of *Aspergillus fumigatus, Candida albicans* and *Lomentospora prolificans* using the primer sets ITS1/FUN_ITS2_1 (left) and ITS1/2 (right).

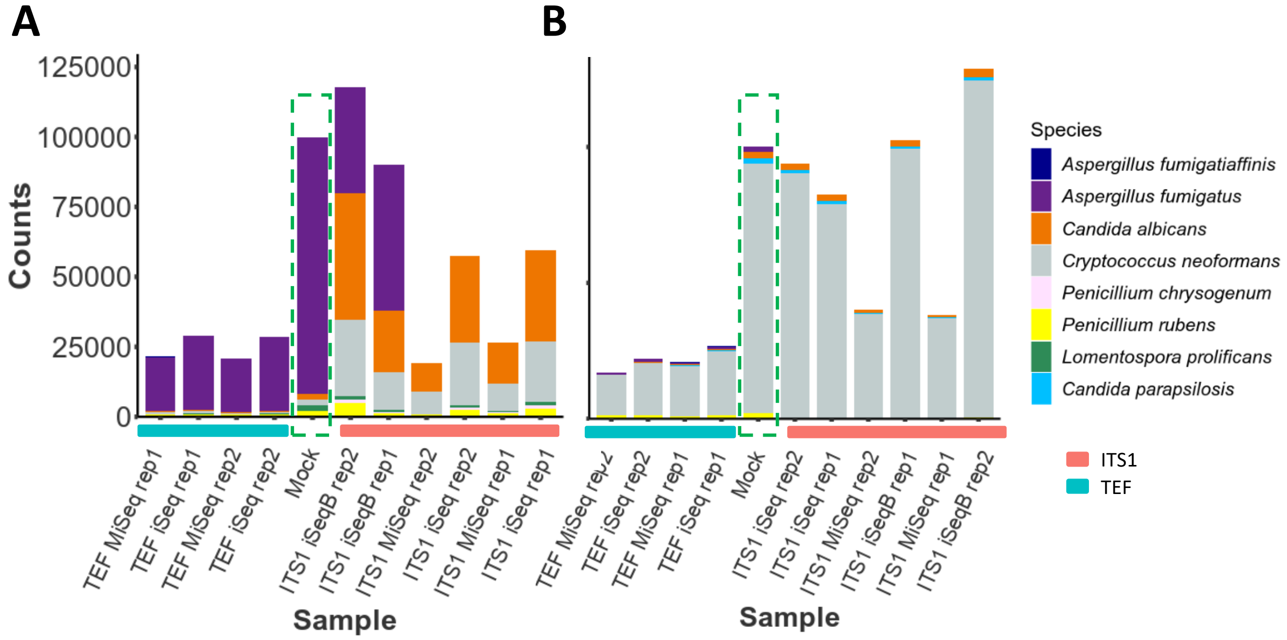

Figure S5. Corresponding raw count data for mock community results in figure 3. Two representative fungal mock community analyses are shown. Each community contained 5 species, was targeted by TEF (A) and ITS1 (B) in duplicate and sequenced on an Illumina iSeq and MiSeq. For ITS1 samples, iSeq sequencing was performed twice. Bar plots indicate raw species count data and expected mock community (dashed green box – arbitrarily set to 100,000 total counts).

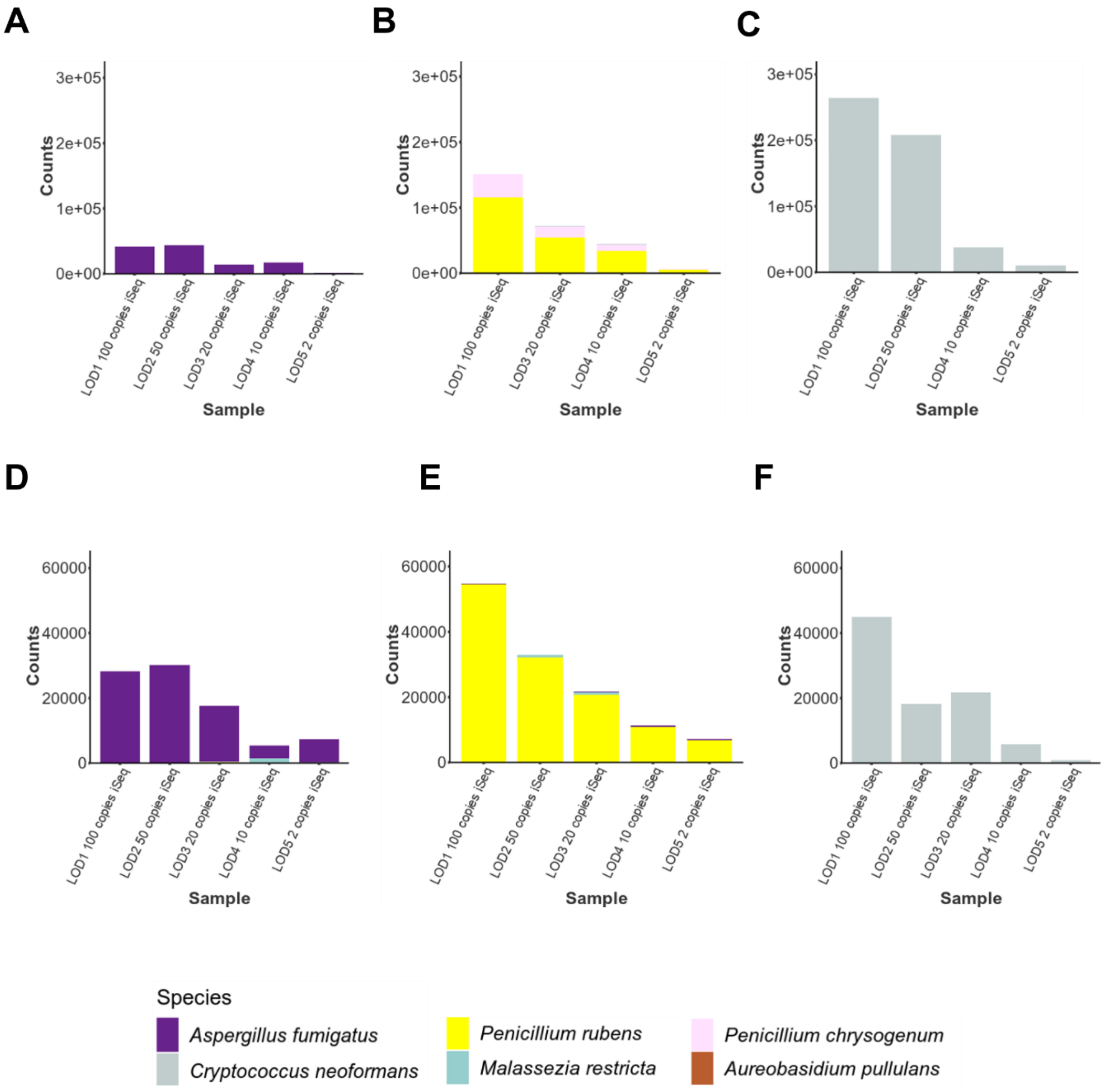

Figure S6. Limit of detection analyses. Bar plots indicate raw species read counts above a 0.2% cutoff. Five haploid genome copy number equivalents of *Aspergillus fumigatus* (A,D), *Penicillium rubens* (B,E) and *Cryptococcus neoformans* (C,F) were each targeted by ITS1 (A-C) and TEF (D-F). In a number of these samples, both targets identified sequences from species which were not present in the sample (false positives). By tracking results from other sequencing runs it was revealed that cross-contamination of barcoded primer sets accounted for these false positives in the TEF assay (D,E) whereas the ITS1 assay could not discriminate between *P. rubens* and *P. chrysogenum* (B) however.

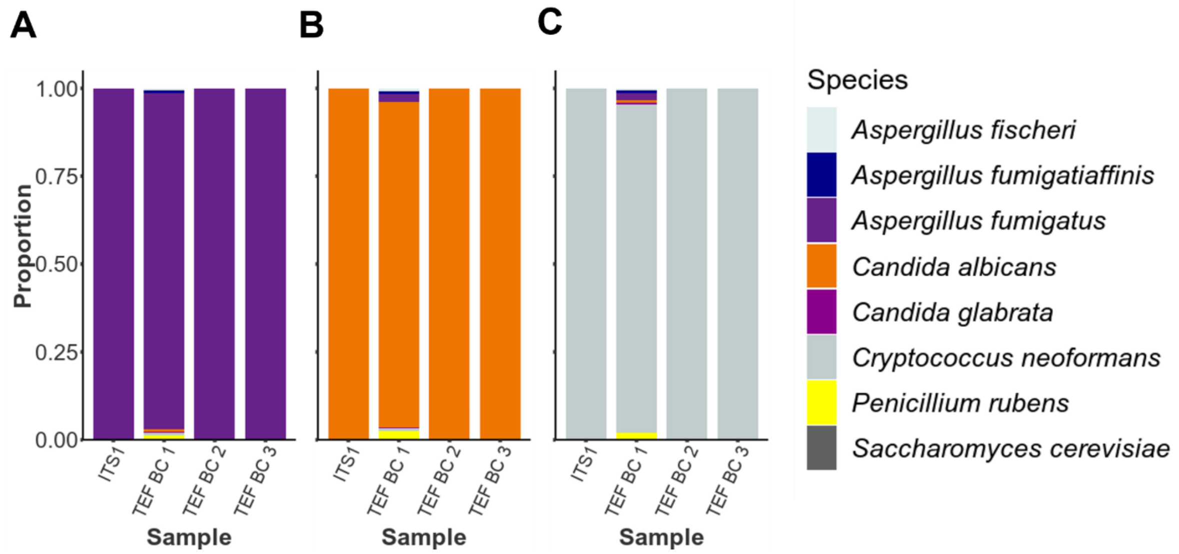

Figure S7. Single species analyses. Bar plots indicate species proportions above a 0.2% cutoff. *Aspergillus fumigatus* (A), *Candida albicans* (B) and *Cryptococcus neoformans* (C) were each targeted by ITS1 and three barcoded TEF primer sets (BC1-3). BC1 primer sets were previously used whereas BC2-3 primer sets contained novel barcode sequences and were previously unused solutions.

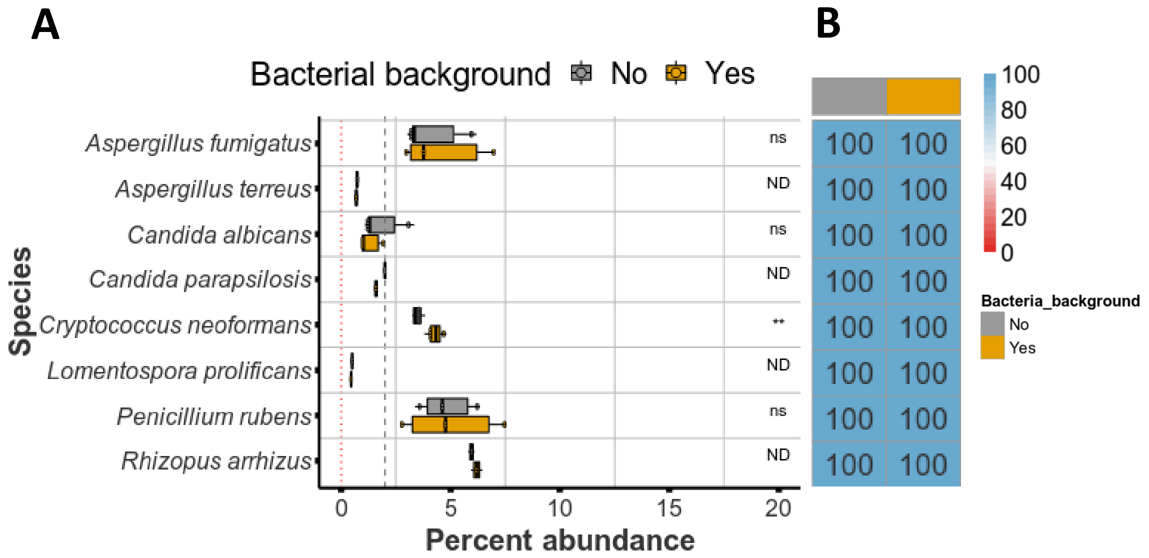

Figure S8. TEF species detection in mock fungal communities is not significantly hindered by bacterial gDNA background. **A.** Boxplot of percent abundance for species spiked at 2% within mock communities targeted by TEF. Expected percent abundance is indicated by dashed black line. Dotted red line indicates no identification. Percent abundances are grouped by bacterial DNA background status (~2000-fold of an *E. coli* and *P. aeruginosa* 50:50 mix). Species level quantifications were significantly different when with and without human background for *C. neoformans* (*; $P < 0.05$ or ***; $P < 0.0001$ by Wilcoxon rank sum test). For all other species which were identified in a sample, quantifications did not differ significantly ($P < 0.05$ by Wilcoxon rank sum test). Wilcoxon rank sum tests were not performed for species with less than 3 data points per human background status and are indicated with 'ND'. **B.** Heatmap representation of TEF identification (ID) rates for species when spiked at 2% within mock communities depending on human DNA background status. Human DNA resulted in no change in detection for all species tested.

A

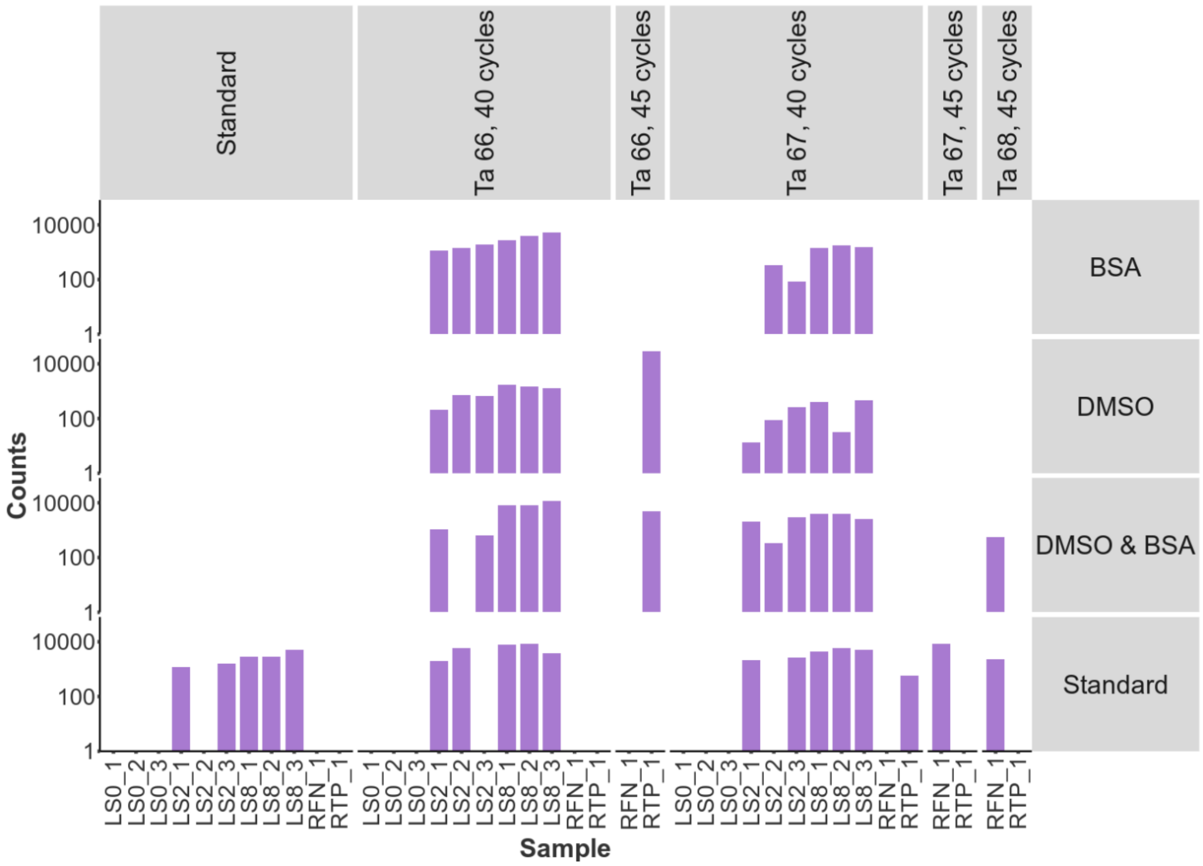

B

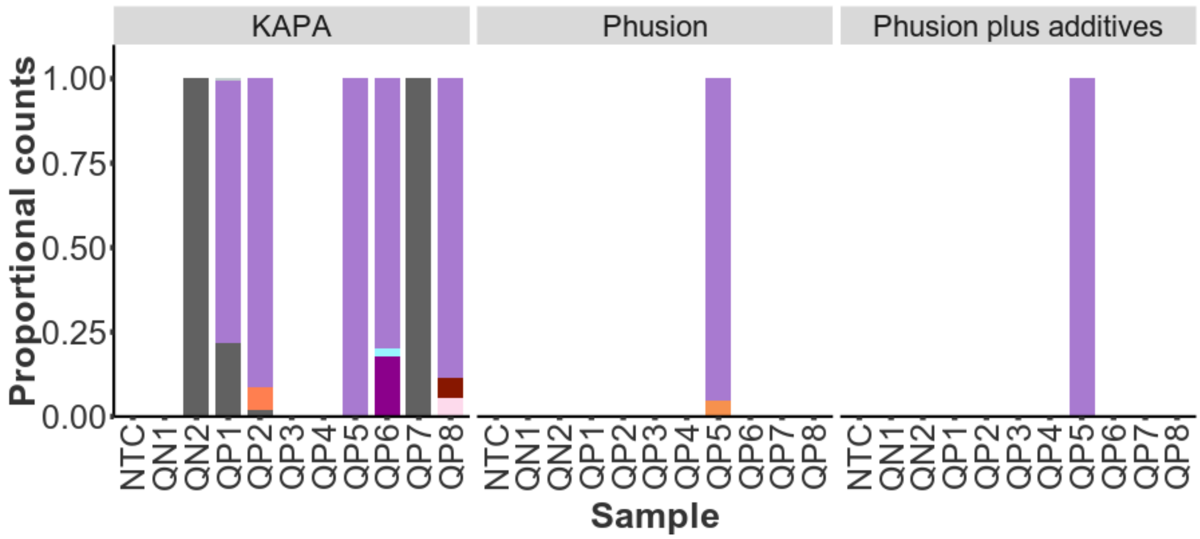

Figure S9. Assay optimisation using clinical respiratory samples. A. Clinical respiratory samples positive (RFN and RTP) or negative (LS0) for *A. fumigatus* were used to optimize the PCR mastermix and cycling parameters. In addition, *A. fumigatus* gDNA spiked samples were used (2 and 8 HE per reaction, LS2 and LS8, respectively). Phusion master mix was used alone, with BSA, DMSO or both BSA and DMSO. Annealing temperatures of 66,67 and 68 °C were tested in combination with 40 or 45 cycles. No template controls were included and produced no fungal reads (not shown). B. Clinical respiratory samples positive (QP1-8) or negative (QN1-2) for *A. fumigatus* were used to compare Phusion, Phusion with additives (BSA and DMSO) and KAPA master mixes.

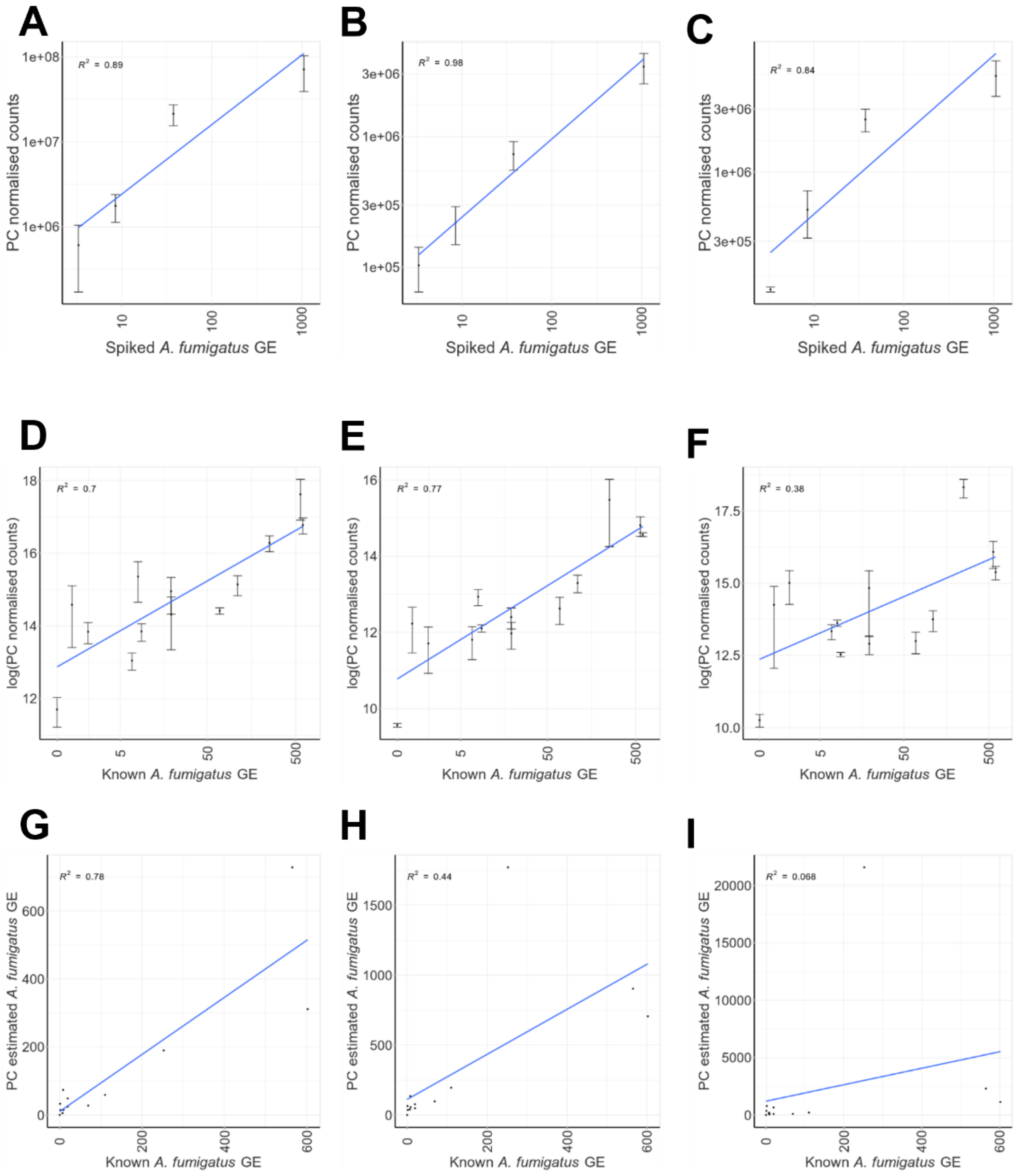

Figure S10. Standard curve method of normalising *A. fumigatus* count data using various internal plasmid controls. A-C. Clinical respiratory samples were spiked with four different amounts of *A. fumigatus* (*A. f*) genome equivalents (GE) to generate a standard curve ranging from ~3 to 1000 GE per PCR. Read counts from the internal plasmid controls (PC1, 4 and 5, respectively) were used to normalize *A. f* counts and positive linear relationships (R^2 > 0.8) with spiked GE were observed for all. D-F. Read counts from the internal plasmid controls (PC1, 4 and 5, respectively) were used to normalize *A. f* counts from 12 clinical respiratory samples known to be positive for *A. f* (plus one negative control sample containing no *A. f*). Normalised counts displayed good correlation with known *A.f* GE (Spearman's rank-order correlation; *r*(11) => 0.7, $p < 0.01$) for two of the three plasmid controls tested. G-I. Using the linear models generated by the standard curve data, *A. f* GE were estimated in the 12 clinical respiratory samples and the negative control. Estimated *A. f* GE for test samples were calibrated using the estimated *A. f* GE value of the negative control sample. The estimated *A. f* GE values displayed strong correlation (Spearman's rank-order correlation; *r*(11) 0.78, $p < 0.01$) with known GE (calculated by rRNA qPCR) when using PC1 only.

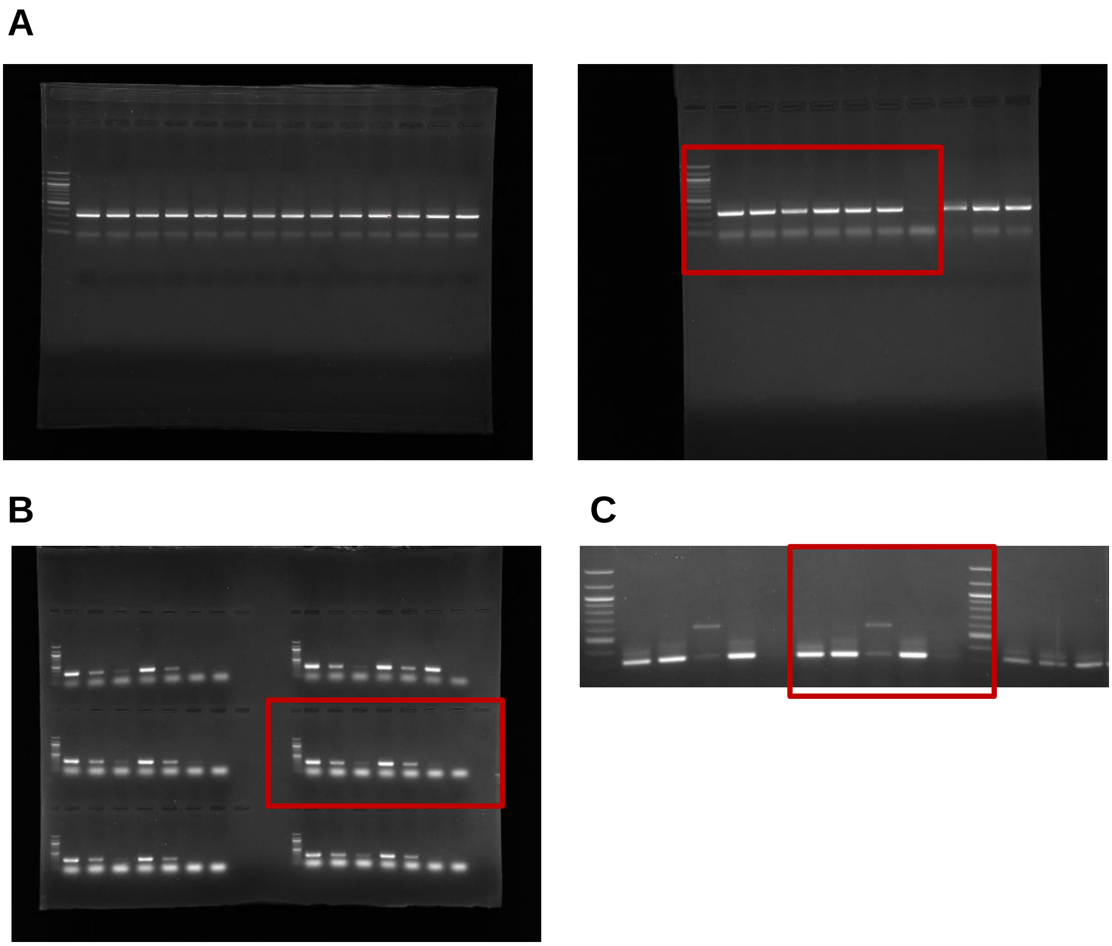

Figure S11. Raw gel images for figure S1. A Raw, uncropped gel images of gel images included in figure S1C. One complete gel (left) was cropped, where one gel (right) was cropped to only include the samples indicated by the red box. Lanes 9, 10 and 11 of this gel (right) contain samples not described in this paper. **B.** Raw, uncropped gel image for the gel image included in figure S1D. The section of gel included in figure S1D are indicated within the red box. All other samples are not described in this paper. **C.** Original gel image for cropped gel image in figure S1E. The section of gel included in figure S1E are indicated within the red box. All other samples are not described in this paper. The full gel image for this gel is not available. The original scan file from the Bio-Rad Gel Doc instrument is available for this image in the supplemental data.
