# Supplementary figures and images for: Development of a novel mycobiome diagnostic for fungal infection
Source: BMC Microbiol. 2024 Feb 19;24:63. doi: 10.1186/s12866-024-03197-5 (PMC10875777; doi:10.1186/s12866-024-03197-5)

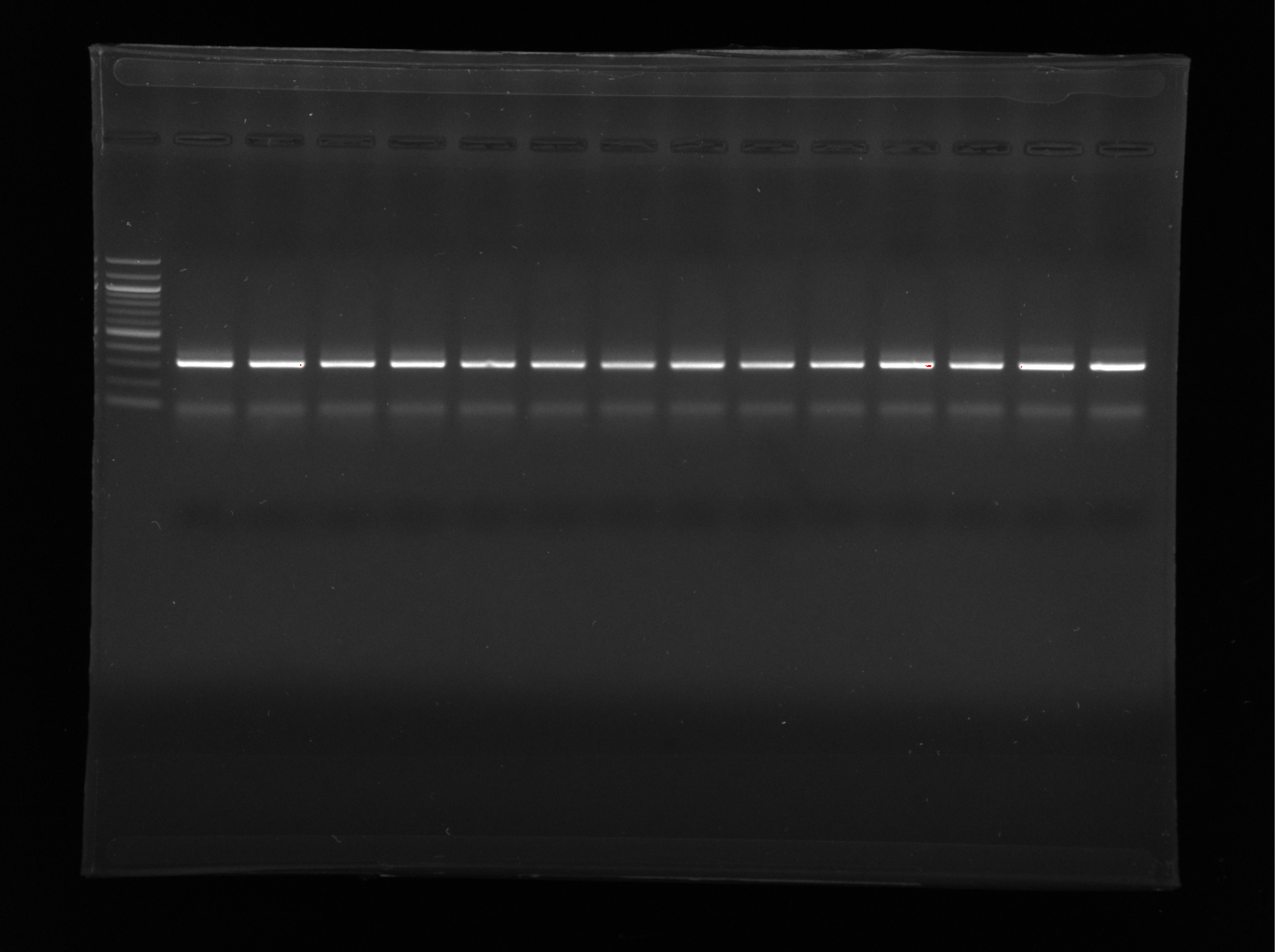

Supplement: Supplementary file 2 — Supplementary Material 2 [file 12866_2024_3197_MOESM2_ESM.png]

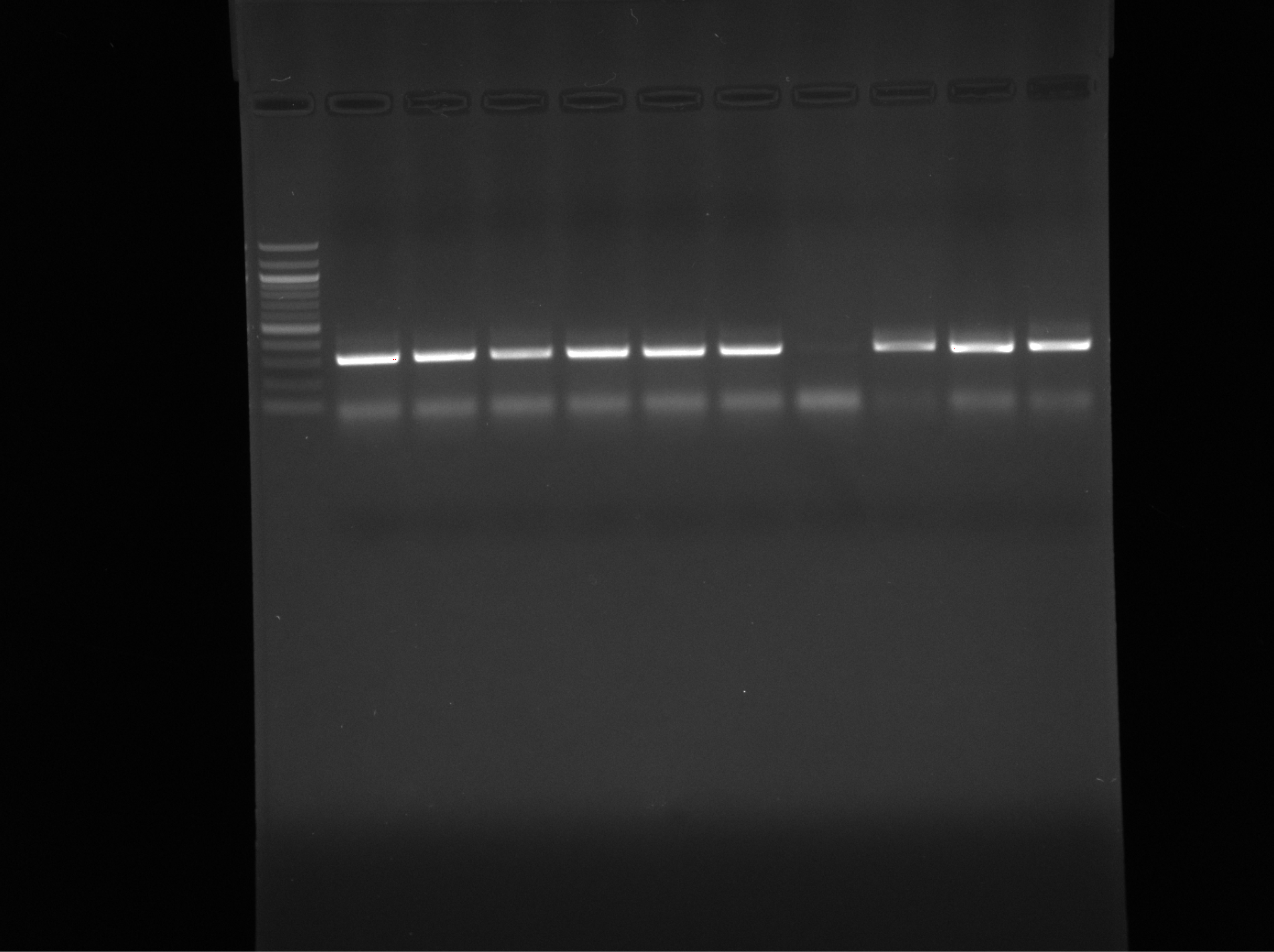

Supplement: Supplementary file 3 — Supplementary Material 3 [file 12866_2024_3197_MOESM3_ESM.png]

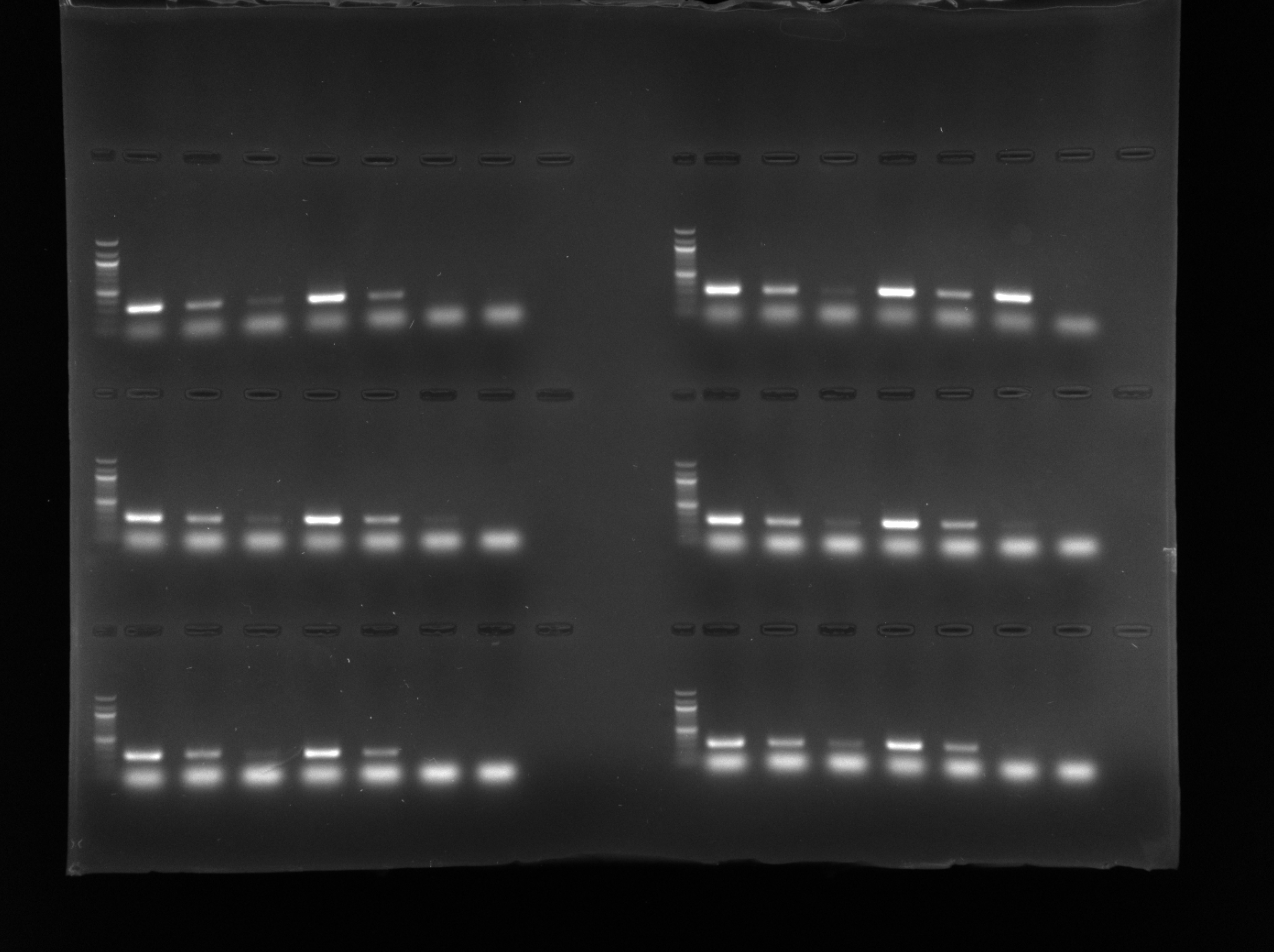

Supplement: Supplementary file 4 — Supplementary Material 4 [file 12866_2024_3197_MOESM4_ESM.png]

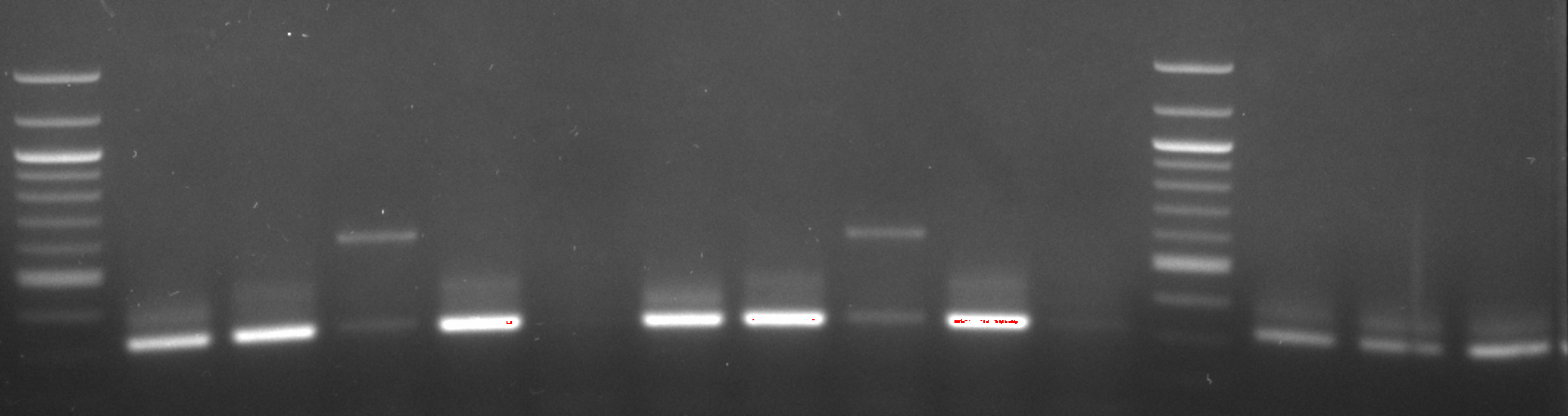

Supplement: Supplementary file 6 — Supplementary Material 6 [file 12866_2024_3197_MOESM6_ESM.png]
